# Supplementary material for: Protocol for a feasibility randomised controlled trial of targeted oxygen therapy in mechanically ventilated critically ill patients
Source: BMJ Open. 2019 Jan 17;9(1):e021674. doi: 10.1136/bmjopen-2018-021674 (PMC6340470; doi:10.1136/bmjopen-2018-021674)
Supplement: Supplementary file 1 [file bmjopen-2018-021674supp001.pdf]

Guidelines for patients in the **INTERVENTION** group of the **TOXYC** study: **T**argeted **OXY**gen therapy in **C**ritical illness

## TARGET $SpO_2$ RANGE: 88 to 92%

Advice on how to maintain your patient's in the target $SpO_2$ range for this study:

| $SpO_2$ | Action |
|---|---|
| > 92% | Reduce $FiO_2$ in 5-10% intervals every 10 minutes until the $SpO_2$ is equal to or less than 92% |
| 88-92% | Maintain $SpO_2$ in target range, reducing or increasing $FiO_2$ in 5% intervals every 10 minutes if required |
| < 88% | Increase $FiO_2$ in 5-10% intervals every 10 minutes until the $SpO_2$ is equal to or greater than 88% |

Other guidance for patients in the **INTERVENTION** group:

- Aim to use the lowest $FIO_2$ possible to achieve the target $SpO_2$.
- Try to avoid excessive use of oxygen prior to interventions such as suctioning. Increasing the $FIO_2$ by approximately 0.25-0.30 (25-30%) briefly should be sufficient in most stable patients.
- Once an $FIO_2$ of 0.21 (21%) has been reached continue to monitor $SpO_2$ but no further downwards titration of $FIO_2$ will be possible.
- Set the $SpO_2$ alarm limits on the monitor to: LOW = **87%**; HIGH = **93%.**
- Do not adjust the $FIO_2$ according to the arterial blood gas $PaO_2$.
- Any mode of ventilation can be used and settings such as the tidal volume, respiratory rate and PEEP can be selected by the patient's clinical team.
- Record all the patient's hourly information in the usual way.
- Please try to minimise unnecessary 100% oxygen boluses and record them all on the ICU chart.

If you have any questions or concerns about the study please contact:
daniel.martin@ucl.ac.uk *or* margaret.mcneil@nhs.net

Thank you for helping us to deliver this study.

*Funded by the National Institute for Health Research and Royal Free Charity*

NHS National Institute for Health Research

royal free charity
