# Supplementary material for: Protocol for a feasibility randomised controlled trial of targeted oxygen therapy in mechanically ventilated critically ill patients
Source: BMJ Open. 2019 Jan 17;9(1):e021674. doi: 10.1136/bmjopen-2018-021674 (PMC6340470; doi:10.1136/bmjopen-2018-021674)
Supplement: Supplementary file 2 [file bmjopen-2018-021674supp002.pdf]

Guidelines for patients in the **CONTROL** group of the **TOXYC** study:
Targeted **OXY**gen therapy in **C**ritical illness

TARGET SpO_2 RANGE: ≥ 96%

Advice on how to maintain your patient's in the target SpO_2 range for this study:

SpO_2	
96-100%	Maintain SpO_2 at or above 96% by increasing or reducing FiO_2 in 5% intervals every 10 minutes if required
< 96%	Increase FiO_2 in 5-10% intervals every 10 minutes until the SpO_2 is equal to or greater than 96%

Other guidance for patients in the **CONTROL** group:

- Once an FiO_2 of 0.21 (21%) has been reached continue to monitor SpO_2 but no further downwards titration of FiO_2 will be possible.
- Set the LOW SpO_2 alarm limit on the monitor to **95%**.
- Do not adjust the FiO_2 according to the arterial blood gas PaO_2, however, if the SpO_2 is consistently at 100%, care must be taken to avoid unnecessary hyperoxaemia.
- Any mode of ventilation can be used and settings such as the tidal volume, respiratory rate and PEEP can be selected by the patient's clinical team.
- Record all the patient's hourly information in the usual way.
- Please record all 100% oxygen boluses on the ICU chart.

If you have any questions or concerns about the study please contact:
daniel.martin@ucl.ac.uk *or* margaret.mcneil@nhs.net

Thank you for helping us to deliver this study.

Funded by the National Institute for Health Research and Royal Free Charity

NHS
National Institute for Health Research

royal free charity
