# Supplementary material for: Protocol for a feasibility randomised controlled trial of targeted oxygen therapy in mechanically ventilated critically ill patients
Source: BMJ Open. 2019 Jan 17;9(1):e021674. doi: 10.1136/bmjopen-2018-021674 (PMC6340470; doi:10.1136/bmjopen-2018-021674)
Supplement: Author's manuscript [file bmjopen-2018-021674.draft_revisions.pdf]

BMJ Open is committed to open peer review. As part of this commitment we make the peer review history of every article we publish publicly available.

When an article is published we post the peer reviewers' comments and the authors' responses online. We also post the versions of the paper that were used during peer review. These are the versions that the peer review comments apply to.

The versions of the paper that follow are the versions that were submitted during the peer review process. They are not the versions of record or the final published versions. They should not be cited or distributed as the published version of this manuscript.

BMJ Open is an open access journal and the full, final, typeset and author-corrected version of record of the manuscript is available on our site with no access controls, subscription charges or pay-per-view fees (http://bmjopen.bmj.com).

If you have any questions on BMJ Open's open peer review process please email info.bmjopen@bmj.com

BMJ Open

Methodology for a feasibility randomised controlled trial of targeted oxygen therapy in mechanically ventilated critically ill patients

Journal:	*BMJ Open*
Manuscript ID	bmjopen-2018-021674
Article Type:	Protocol
Date Submitted by the Author:	17-Jan-2018
Complete List of Authors:	Martin, Daniel S.; University College London, Division of Surgery and Interventional Science<br>Brew-Graves, Chris; Division of Surgery and Interventional Science, University College London<br>McCartan, Neil; University College London, Surgical & Interventional Trials Unit<br>Jell, Gavin; University College London, Division of Surgery and Interventional Science<br>Potyka, Ingrid; University College London<br>Stevens, Jia; University College London, Division of Surgery and Interventional Sciences<br>Williams, Norman; University College London<br>McNeil, Margaret; Royal Free Hospital, Critical Care Unit<br>O'Driscoll, Ronan; Salford Royal NHS Foundation Trust, Respiratory medicine<br>Mythen, Monty; University College Hopital, Anaethesia<br>Grocott, Michael P. W.; University of Southampton,
Keywords:	Adult intensive & critical care < ANAESTHETICS, oxygen, hypoxia, artificial respiration, respiratory insufficiency, oxidative stress

SCHOLARONE™
Manuscripts

TITLE: Methodology for a feasibility randomised controlled trial of targeted oxygen therapy in mechanically ventilated critically ill patients

AUTHORS: * Daniel Martin[1,2], Chris Brew-Graves[3], Neil McCartan[3], Gavin Jell[2], Ingrid Potyka[3], Jia Stevens[1,2], Norman R Williams[3], Margaret McNeil[1], B Ronan O'Driscoll[4], Monty Mythen[5], Michael Grocott[6,7]

AFFILIATIONS:

1. Critical Care Unit, Royal Free Hospital, Pond Street, London, NW3 2QG

2. Division of Surgery and Interventional Science, Royal Free Campus, University College London, Royal Free Hospital, Pond Street, London, NW3 2QG

3. Surgical & Interventional Trials Unit (SITU), University College London Division of Surgery & Interventional Science, 3rd Floor, Charles Bell, House, 43 – 45 Foley Street, London, W1W 7JN

4. Manchester Academic Health Sciences Centre, and Salford Royal Foundation NHS Trust Stott Lane, Salford M6 8HD

5. Anaesthesia and Critical Care, University College London Hospitals National Institute of Health Research Biomedical Research Centre, 170 Tottenham Court Road, London, W1T 7HA

6. Integrative Physiology and Critical Illness Group, Clinical and Experimental Sciences, Faculty of Medicine, University of Southampton, Southampton, UK

7. Critical Care Research Group, Southampton NIHR Biomedical Research Centre, University Hospital Southampton, Southampton, UK

* Corresponding author: Dr Daniel Martin: Critical Care Unit, Royal Free Campus, University College London, Royal Free Hospital, Pond Street, London, NW3 2QG. +44 (0)20 7794 0500 (RFH ext 39481). daniel.martin@ucl.ac.uk

For peer review only

For peer review only - http://bmjopen.bmj.com/site/about/guidelines.xhtml

KEYWORDS: Oxygen; critical care; hypoxia; artificial respiration; respiratory insufficiency; oxidative stress

For peer review only

For peer review only - http://bmjopen.bmj.com/site/about/guidelines.xhtml

ABSTRACT

Introduction: Oxygen is the most commonly administered drug to mechanically ventilated critically ill adults, yet little is known about the optimum oxygen saturation (SpO_2) target for these patients; the current standard of care is an SpO_2 of 96% or above. Small pilot studies have demonstrated that permissive hypoxaemia (aiming for a lower SpO_2 than normal by using a lower fractional inspired oxygen concentration (F_IO_2)) can be achieved in the critically ill and appears to be safe. This approach has not been evaluated in a National Health Service setting. It is possible that permissive hypoxaemia may be beneficial to critically ill patients thus it requires robust evaluation.

Methods and analysis: Targeted oxygen therapy in critical illness (TOXYC) is a feasibility randomised controlled trial (RCT) to evaluate whether recruiting patients to a study of permissive hypoxaemia is possible in the UK. It will also investigate biological mechanisms that may underlie the links between oxygenation and patient outcomes. Mechanically ventilated patients with respiratory failure will be recruited from critical care units at two sites and randomised (1:1 ratio) to an SpO_2 target of either 88-92% or ≥ 96% whilst intubated with an endotracheal tube. Clinical teams can adjust F_IO_2 and ventilator settings as they wish to achieve these targets. Clinical information will be collected before, during and after the intervention and blood samples taken to measure markers of systemic oxidative stress. The primary outcome of this study is feasibility, which will be assessed by recruitment rate, protocol adherence, and withdrawal rates. Secondary outcomes will include a comparison of standard critical care outcome measures between the two intervention groups, and the measurement of biomarkers of systemic oxidative stress. The results will be used to calculate a sample size, likely number of sites, and overall length of time required for a subsequent large multicentre RCT.

For peer review only - http://bmjopen.bmj.com/site/about/guidelines.xhtml

Ethics and dissemination: This study was approved by the London - Harrow Research Ethics Committee on 2nd November 2017 (REC Reference 17/LO/1334) and received HRA approval on 13th November 2017. Results from this study will be disseminated in peer reviewed journals, the NIHR Journals Library and patient information websites.

The study has been funded by the National Institute of Health Research and Royal Free Charity. It is registered on www.clinical trial.gov (NCT03287466).

For peer review only

STRENGTHS AND LIMITATIONS OF THIS STUDY

- This is the first study of permissive hypoxaemia in critically ill patients in a National Health Service setting
- It is a small randomised controlled trial to assess feasibility, not the efficacy of the intervention
- The study will compare levels of biomarkers of systemic oxidative stress between the two intervention groups
- It will provide valuable information to enable the design of future large-scale randomised controlled trials

For peer review only - http://bmjopen.bmj.com/site/about/guidelines.xhtml

INTRODUCTION

In the UK there are over 190,000 admissions to adult critical care units each year (www.icnarc.org). Approximately 40% of these patients will require mechanical ventilation and the mortality rate in this group is approximately 30% [1,2]. Hypoxaemia is common amongst this cohort of patients and we lack evidence-based guidelines for their management, particularly regarding what levels of arterial oxygenation are acceptable or optimal. It has been proposed that attempting to fully reverse hypoxaemia in critically ill patients may pose a greater risk of harm than allowing moderate hypoxaemia to persist, a concept called permissive hypoxaemia [3]. The premise behind permissive hypoxaemia is that the interventions used to correct hypoxaemia may themselves cause harm, in particular high concentrations of inspired oxygen, therefore safely minimising their use could be beneficial [4–7].

Oxygen has the potential to cause harm when used in high concentration, primarily via its toxic effect on the lungs [8–10]. Reactive oxygen species (ROS) released mainly from the inner mitochondrial membrane during oxidative phosphorylation serve an essential role in cellular signalling but in excess these highly reactive molecules are able to destroy lipids, proteins and DNA. Their rate of release is determined by cellular oxygen tension [11] and the extent of damage caused by them can be measured by evaluating biomarkers of tissue degradation [12]. The lung parenchyma is particularly susceptible to oxygen toxicity in critically ill patients as a result of being exposed to high concentration oxygen during mechanical ventilation. The threshold above which harm may be caused (in terms of concentration and duration of exposure) in critically ill patients is unclear, but since lung injury is common in this patient cohort the threshold may well be lower than in other patients or healthy volunteers. During critical illness the propagation of pro-inflammatory pathways, with the activation of leukocyte and vascular endothelial responses further increase the ROS burden [13]. This depletes endogenous antioxidants, which normally regulate ROS homeostasis [14]. As a consequence, oxidative stress is a key mechanism of injury

For peer review only - http://bmjopen.bmj.com/site/about/guidelines.xhtml

in systemic multi-organ failure, and has been linked to increase in morbidity and mortality in critical illness [15,16].

Oxygen is a drug a relatively narrow therapeutic index. It should therefore be prescribed, administered and monitored in a manner comparable to other drugs that have toxic side effects. There appears, however, to be wide variation in practice regarding its use and opinions about oxygenation in the critically ill [17,18]. This is perhaps the result of a paucity of evidence from robust clinical trials; a somewhat surprising situation given that almost every patient admitted to a critical care unit will receive supplementary oxygen. The traditional teaching that hypoxaemia must be avoided at all costs, may have led to a disregard to the potential harm caused by excessive oxygen, and this requires evaluation.

A small number of studies have begun to explore permissive hypoxaemia as a viable treatment strategy in the critically ill, primarily assessing feasibility and safety. In the first study of its kind, 105 mechanically ventilated patients were assessed in a before (n=51) and after (n=54) design [19]. Following a period of standard oxygenation practice in a single centre (aiming for normal or high blood oxygen levels), a practice change was initiated in which oxygen saturation (SpO_2) was maintained at 90–92%. The authors of this study concluded that the conservative oxygen therapy intervention was feasible and free of adverse biochemical, physiological, or clinical outcomes. A comparable strategy was used on a much larger scale in a two-stage model, moving from normal oxygenation to an SpO_2 of 92–95% [20]. These authors reported that mechanical ventilation time was significantly lower during both study (lower SpO_2) phases compared to baseline. The adjusted ICU mortality and ICU-free days did not significantly differ between study phases but mortality decreased in reference to baseline for both of the low SpO_2 phases. In the first multicentre randomised controlled trial (RCT) of permissive hypoxaemia a

For peer review only - http://bmjopen.bmj.com/site/about/guidelines.xhtml

total of 103 mechanically ventilated patients were allocated to either a conservative oxygenation group (SpO_2 of 88–92%) or a liberal oxygenation group (SpO_2 of greater than or equal to 96% [21]. The purpose of the study was to confirm feasibility and this was demonstrated, along with no obvious signs of harm in the low SpO_2 group. The most recently published trial of permissive hypoxaemia was a single centre RCT that compared SpO_2 targets of 94%-98% versus 97-100% [22]. The primary outcome of this study was ICU mortality, and the values reported were: 11.6% in the conservative group and 20.2% in the conventional group, giving an absolute risk reduction of 8.6% (1.7-15.0%). This study had a number of limitations [23] but still adds weight to the argument that permissive hypoxaemia appears to not be harmful and may be of benefit.

METHODS

The trial was designed according to the SPIRIT (Standard Protocol Items: Recommendations for Interventional Trials) statements [24].

Trial aim and objectives

The Targeted OXYgen therapy in Critical illness (TOXYC) study aims to determine whether reducing the SpO_2 target in patients requiring mechanical ventilation is feasible (in terms of participant recruitment and delivery of the intervention) in a National Health Service (NHS) setting. In doing this we hope to inform future investigators who wish to construct larger trials in this field. The objectives are to construct a randomised controlled trial of conventional oxygenation versus permissive hypoxaemia, identify any potential barriers to research in this field and explore biological mechanisms that may explain the proposed benefits from the intervention. The project was favourably supported at the UK Critical Care Research Forum in 2016.

For peer review only

For peer review only - http://bmjopen.bmj.com/site/about/guidelines.xhtml

Primary outcome measure

The primary outcome of this study is feasibility, which will be assessed in the following ways: i) the ability to recruit patients (recruitment rate), ii) protocol deviations, iii) rate of withdrawal from the study (in both the intervention and control groups), and iv) the reasons for any withdrawal from the study.

Feasibility of recruitment will be evaluated by monitoring patient screening and subsequent agreement to participate, along with any withdrawal of consent during or after the study. Implementation of the study protocol will be evaluated by analysing adherence to oxygenation targets and completion of the treatment without protocol deviations. Reasons for withdrawal will be assessed by the trial management group at the end of the study to assess whether there are common themes that can be addressed in the future.

Secondary outcome measures

Measurements of oxidative stress (including 4- hydroxynonenal, protein carbonyls, total antioxidant capacity and glutathione reductase) will be made in blood samples taken from participants to understand the potential biological mechanisms that link blood oxygen levels to clinical outcomes. In addition, routine clinical data and outcome measures will be collected from the participants to assess the safety of the intervention. Finally, length of critical care stay, length of hospital stay, and survival at critical care unit discharge, 30 and 90 days will be collected. This information will be essential for the design of future larger trials.

Trial design

TOXYC, a multi-centre RCT, which will be conducted at two sites, is a trial of targeted oxygen therapy in adult critically ill patients receiving mechanical ventilation via an endotracheal tube. Sixty patients will be allocated on a 1:1 basis to either a normal SpO_2 target group or a lower than normal SpO_2 target group. A flow diagram of the study is shown in Figure 1.

For peer review only - http://bmjopen.bmj.com/site/about/guidelines.xhtml

Selection of participants

Screening

Screening will occur as part of routine research activity on the two critical care units involved in the study. Research nurses will use medical notes to determine initial suitability for the study, according to the inclusion and exclusion criteria. No additional tests or examinations will be required to ascertain whether patients are eligible for the study. Screening will occur as patients are admitted to the critical care units to minimise the time from admission to enrolment.

Inclusion criteria

- Unplanned admission to a critical care unit
- 18 years of age and above (no upper age limit)
- Respiratory failure forms part of the admission diagnosis
- The patient is mechanically ventilated via an endotracheal tube
- The patient is expected to receive mechanical ventilation for > 72 hours

Exclusion criteria

- Admission following surgery (elective or unplanned)
- Those patients expected to die within 24 hours of admission to ICU
- Pregnant females
- Admission post-cardiac arrest
- Patients with chronic lung disease known (or highly suspected) to have baseline oxygen saturations in the range of the intervention arm (i.e. 88-92%)
- Admission post trauma (including traumatic brain injury)
- Known sickle cell trait or disease
- Ongoing significant haemorrhage or profound anaemia

For peer review only

- Severe peripheral vascular disease
- Severe pulmonary hypertension
- Other medical conditions where mild hypoxaemia would be contra-indicated
- Patients participating in other interventional clinical trials

Enrolment

Consent

Due to the severity of illness of the patients being recruited to this study, and the use of sedative drugs that are required for mechanical ventilation, it is unlikely that potential participants will have capacity. Should a potential participant be deemed to have capacity they will be approached by the research team, given a patient information sheet (PIS) and then provided with an opportunity to ask questions. After an appropriate length of time, the research team will seek informed consent from the patient if they wish to participate.

If the patient lacks capacity to provide informed consent, a Personal Consultee (PeC) will be appointed to represent them. This could be the patient's next of kin, a relative or close friend with whom to discuss the patient's participation in the trial. The research team will seek the PeC's opinion as to whether patient would wish to take part in the trial, providing for them an appropriate version of the ethically approved PIS. If the PeC believes the patient would have wanted to participate in the study (or would not have objected to it), they will be asked to sign a PeC agreement form. If there is no PeC present or immediately available in person, opinion may be sought from a suitable person via the telephone and then a telephone agreement form completed by a member of the research team.

If there is no identifiable PeC for a potential participant then they will be provided with a Professional Consultee (PrC), who is completely independent of the study. Their opinion will be sought as to whether it is appropriate for the patient to be enrolled into the study. Opinion will be

For peer review only - http://bmjopen.bmj.com/site/about/guidelines.xhtml

sought in the same manner as for the PeC, involving the appropriate version of the ethically approved PIS.

Adequate time will be given for consideration by the patient or PeC/PrC to consider the information in the PIS and ask questions. The research team will record when the PIS has been given to the patient or their consultee. Due to the nature of this patient cohort (critically ill patients requiring substantial organ support due to the severity of their illness) the length of time from identifying a potential participant to initiating the intervention is likely to be less than 24 hours. This is to avoid dilution of the intervention or control effect prior to its commencement.

If a participant who lacked capacity at the point of recruitment subsequently becomes able to provide informed consent (because they gain capacity on recovery from their illness), they will be informed about their participation in the study, provided with a PIS and asked whether they would be willing to provide retrospective consent. At this point the participant will be given the opportunity to withdraw from the study and to decide if the data (and blood samples) collected from them can be included in the final analysis.

All patient consent and consultee agreement procedures will adhere to the Mental Capacity Act (2005).

Randomisation

Randomisation will be carried out online after a patient has been recruited to the study and an agreement form or consent form has been signed. It will be conducted in a 1:1 manner for the intervention and control groups, stratified by study site, using random permuted blocks of different size. The process of randomisation will be conducted online (www.sealedenvelope.com).

For peer review only

Withdrawal

Participants will be withdrawn from the study if:

- The responsible clinician deems it inappropriate for treatment to continue due to a change in the patient's condition
- The Chief Investigator or delegated member of the research team deems it inappropriate for treatment to continue due to a change in the patient's condition
- Agreement for the patient to participate in the study is withdrawn by the PeC or PrC
- The patient regains capacity and choses to withdraw from the study

As this is a feasibility study, patients withdrawn from the study will not be replaced, but the reason for withdrawal will be recorded.

Trial treatment

Intervention

The intervention is a more conservative use of oxygen via the ventilator to achieve an SpO_2 of 88-92%, lower than normal practice in most critical care units in the UK. The intervention will be delivered by the participant's clinical team, which will consist of the critical care doctors and nurses at the two study centres. These teams will be provided with guidance to help keep participants within their target SpO_2 (*supplementary material 1*) but this will not be protocolised.

Comparator

The control group will receive oxygen to maintain an SpO_2 at 96% or above (standard care). As per the intervention group, guidance will be provided to the clinical team to help maintain participants within their target SpO_2 (*supplementary material 2*).

For peer review only - http://bmjopen.bmj.com/site/about/guidelines.xhtml

Duration of treatment

The aim is for the intervention to be commenced as soon as possible after admission to the critical care unit (following enrolment) and end following removal of the participant's endotracheal tube. Specific treatment end-points for both groups would therefore include i) extubation, ii) formation of a tracheostomy, iii) transfer to another critical care unit, and iv) death. The research team will review enrolled participants daily to monitor adherence to SpO_2 targets and provide bedside advice where required. No targets or limitations will be set for arterial partial pressure of oxygen (PaO_2) or carbon dioxide ($PaCO_2$).

Standard clinical management.

Aside from the designated SpO_2 targets, all other aspects of care will remain the same between the intervention and control groups. Regular arterial blood gases should be taken during the trial period, according to local clinical guidelines; no additional arterial blood gases will be necessary for the purpose of the study.

Data collection

Data will be collected from various sources including the participant's medical records, bedside charts and hospital computer systems. Data will be collected from these primary sources by members of the research team and entered into an electronic clinical record form (eCRF).

Baseline data collection

- Patient demographics: age, gender, height and weight
- Cause of respiratory failure (diagnosis)
- The presence of any chronic diseases
- Acute Physiology and Chronic Health Evaluation (APACHE) II score (and its components)
- Sequential Organ Failure Assessment (SOFA) score (and its components)
- *Respiratory measurements*: PaO_2, $PaCO_2$, pH, SpO_2, FIO_2, ventilator settings and measures

For peer review only - http://bmjopen.bmj.com/site/about/guidelines.xhtml

- *Cardiovascular measurements*: blood pressure, heart rate, cardiac rhythm, vasopressor / inotrope dose, fluid balance
- *Renal measurements*: creatinine, urine output in past 24 hours, the need for renal replacement therapy
- *Hepatic measurements*: transaminases, blood clotting values and bilirubin
- Blood lactate concentration

Subsequent data collection during treatment

- Most measures will be taken daily, except for those specifically related to oxygenation, which will be collected hourly, to permit detailed analysis of compliance to blood oxygenation target.
- Time to extubation or detachment from mechanical ventilation, and mechanical ventilation free days on ICU
- Adverse events occurring during the study period

Follow up

- Length of ICU stay
- Length of hospital stay
- 30 and 90 day survival rates, and days alive out of hospital
- Adverse events

Data Management

This trial will use an eCRF and trial data will be entered into an approved, protected database (https://www.elsevier.com/solutions/macro). Access to the eCRF system will only be provided to staff with relevant authority. Participants will be given a unique subject number and subject identifier. Data will be entered under this identification number onto the central database stored

For peer review only

on the servers. The database will be password protected and only accessible to members of the TOXYC study team and external regulators if requested. At site, access will only be granted to staff with permission on the delegation log, and after training. The servers are protected by firewalls and are patched and maintained according to best practice. The physical location of the servers is protected by CCTV and security door access. The database software provides a number of features to help maintain data quality, including; maintaining an audit trail, allowing custom validations on all data, allowing users to raise data query requests, and search facilities to identify validation failure/ missing data.

The identification, screening and enrolment logs, linking participant identifiable data to the pseudo-anonymised subject numbers will either be held in written form in a locked filing cabinet or electronically in password protected form on hospital computers. After completion of the study the identification, screening and enrolment logs will be stored securely by the sites for 20 years. After completion of the study the identification, screening and enrolment logs will be stored securely by the sites for 20 years.

Sample collection, storage and processing

Blood samples will be taken from participants in order to evaluate oxidative stress. Samples will be taken from an indwelling arterial catheter that is already present in the patient as part of routine critical care. Blood will be processed at each of the two centres according to a defined standard operating procedure, and then stored at -80 degrees centigrade. These blood samples will be taken at baseline (shortly after the patient has been recruited into the study but prior to their treatment being commenced), and on days 2, 3, 5 and 10 after recruitment. A number of biomarkers of oxidative stress will be measured, including: 4- hydroxyl-2-nonenal, protein carbonyls, total antioxidant capacity and glutathione reductase.

For peer review only - http://bmjopen.bmj.com/site/about/guidelines.xhtml

Safety monitoring

All adverse events will be recorded in the medical records and reported to the appropriate body; either online to SITU or by email to the sponsor. Table 1 shows a basic list of expected adverse events that will be recorded within the patient's eCRF and medical notes, but the sponsor will not be informed. Adverse events (AEs) and serious adverse events (SAEs) will be summarised descriptively by interventional group at the end of the study.

For peer review only

**RESPIRATORY**	**CARDIOVASCULAR**	**HAEMATOLOGICAL**
• reintubation<br>• arterial desaturation<br>• pneumothorax<br>• pleural effusion<br>• pneumonia<br>• pulmonary embolism	• arrhythmia<br>• hypotension<br>• requirement for inotropic support	• Anaemia<br>• Low platelet count<br>• High white blood cell count
**RENAL**	**GASTROINTESTINAL**	**NEUROLOGICAL**
• Acute kidney injury<br>• Requirement for renal support<br>• hyperkalaemia	• Diarrhoea<br>• vomiting<br>• Failure to absorb enteral feed	• Delirium / agitation

Table 1. A list of expected adverse events that may occur during the course of the study

For peer review only - http://bmjopen.bmj.com/site/about/guidelines.xhtml

Trial monitoring and oversight

The TOXYC trial will report to a data monitoring committee, and a trial steering committee will be appointed to provide study oversight on behalf of the sponsor and funder. Day to day management of the trial will be the responsibility of the trial coordinator with oversight from the trial management group.

Statistics

No formal comparative analyses are planned in this feasibility study. The primary and secondary outcome measures will be presented using summary statistics (e.g. means, standard deviations, medians, proportions). Missing data, non-compliers and withdrawals will be looked at in detail to determine if there is any evidence of bias.

A CONSORT diagram will be completed, summarising the number of patients eligible for the study, the number randomised in each arm, and enumerating in detail those not approached (with reasons), the number of withdrawals (with reasons) overall and per arm. Recruitment rate (overall, per site, and peak recruitment rate per month) will be determined. Monthly and cumulative accrual graphs will be constructed. Baseline characteristics of randomised participants will be summarised, including gender, age, height, weight, details of medical history, pre-intervention APACHE II and SOFA scores. Mean and SD, or median and IQR, will be calculated as appropriate. For the secondary objectives, summary statistics on respiratory, cardiovascular, renal, and hepatic measurements will be calculated at the appropriate time points (hourly or daily). Length of stay in the critical care unit and in hospital will be summarised. 30 and 90 day mortality rates will be calculated, and "days alive and out-of-hospital" determined for each patient and summarised using appropriate measures. Compliance will be assessed. For each patient, the proportion of time spent within the randomisation-determined oxygen

For peer review only - http://bmjopen.bmj.com/site/about/guidelines.xhtml

saturation limits will be calculated, and summarised by treatment arm. Adverse events will be tabulated and grouped according to seriousness, severity and causality.

All variables will be checked for completeness and checked for the presence of outliers. Graphical depictions of results will be prepared, both on a per-patient basis (especially for the longitudinal data such as hourly oxygen measurement) and grouped by intervention. Frequency distribution curves will be shown where appropriate (especially for the "length of stay" measurements).

No hypothesis testing is envisaged for this feasibility study. The results will be used to calculate a sample size, likely number of sites, and overall length of time required for a subsequent large multicentre RCT.

Ethical compliance

This Trial was approved by the London - Harrow Research Ethics Committee on 02NOV17 (REC Reference 17/LO/1334) and received HRA approval on 13NOV17. TOXYC is also registered on www.clinical trial.gov (NCT03287466)

CONCLUSION

The results of this feasibility trial will inform researchers about the ability to conduct a study evaluating permissive hypoxaemia in critically ill patients. It will provide information about recruitment rates in UK critical care units and help to identify any barriers to future research. Furthermore, results from oxidative stress marker analysis may highlight biological markers of importance in the pathway between oxygen administration and patient outcomes.

For peer review only

Funding statement: This work was supported by the National Institute for Health Research (PB-PG-0815-20006) and Royal Free Charity.

Authors' contributions:

DM: Conceived idea, designed study, obtained funding for RCT, obtained funding for mechanistic component, obtained ethics approval, will be CI for study and PI at Royal Free, wrote manuscript, revised manuscript

CB-G: obtained ethics approval, responsible for trial governance, wrote manuscript, revised manuscript

NM: obtained ethics approval, responsible for trial governance, revised manuscript

GJ: obtained funding for mechanistic component, lead for biochemical analysis, revised manuscript

IP: responsible for data collection, revised manuscript

JS: involved in sample processing, revised manuscript

NW: obtained ethics approval, responsible for trial governance, responsible for data analysis and statistics, wrote manuscript, revised manuscript

MMcN: lead research nurse for study, responsible for screening patients and collecting data, revised manuscript

BRO'D: obtained funding for RCT, involved in study design, revised manuscript

MM: Conceived idea, designed study, obtained funding for RCT, revised manuscript

MG: Conceived idea, designed study, obtained funding for RCT, PI at Southampton General Hospital, wrote manuscript, revised manuscript

Competing interests statement:

For peer review only - http://bmjopen.bmj.com/site/about/guidelines.xhtml

DM, MM and MG are directors of a company developing an oxygen delivery device (Oxygen Control Ltd).

DM has received honoraria and consultancy fees from Siemens Healthcare, Masimo, Deltex and Edwards Lifesciences.

MG is the National Specialty Lead for Anaesthesia, Perioperative Medicine and Pain within the UK National Institute of Heath Research Clinical Research Network, an elected council member of the Royal College of Anaesthetists and serves on the board of the Evidence Based Perioperative Medicine (EBPOM) social enterprise and the medical advisory board of Sphere Medical Ltd. MG has received honoraria for speaking and/or travel expenses from Edwards Lifesciences, Fresenius-Kabi, BOC Medical (Linde Group), Ely-Lilly Critical Care, and Cortex GmBH. MG is executive chair of the Xtreme-Everest Oxygen Research Consortium and joint Editor-in-Chief of the journal *Perioperative Medicine.*

MM is a consultant for Baxter, Edwards Lifesciences and Deltex; his University Chair is supported by Smiths Medical; Elected Council Member Royal College of Anaesthetsists; Editorial Board BJA and Critical Care; Founding Editor-in-Chief Perioperative Medicine.

JS, NW, ROD, GJ and MMcN have no competing interests.

Contributions: DM, CB-G, NM, GJ, IP, JS, NW, MMc, BRO'D, MM & MG conceived and designed the study. DM wrote the manuscript and all authors contributed to revising it to its final format.

For peer review only

REFERENCES

1. Eastwood G, Bellomo R, Bailey M et al. Arterial oxygen tension and mortality in mechanically ventilated patients. *Intensive Care Med* 2012; **38**: 91-98
2. de Jonge E, Peelen L, Keijzers PJ et al. Association between administered oxygen, arterial partial oxygen pressure and mortality in mechanically ventilated intensive care unit patients. *Crit Care* 2008; **12**: R156
3. Martin DS, Grocott MP. Oxygen therapy in critical illness: precise control of arterial oxygenation and permissive hypoxemia. *Crit Care Med* 2013; **41**: 423-432
4. Abdelsalam M, Cheifetz IM. Goal-directed therapy for severely hypoxic patients with acute respiratory distress syndrome: permissive hypoxemia. *Respir Care* 2010; **55**: 1483-1490
5. Capellier G, Panwar R. Is it time for permissive hypoxaemia in the intensive care unit? *Crit Care Resusc* 2011; **13**: 139-141
6. He HW, Liu DW. Permissive hypoxemia/conservative oxygenation strategy: Dr. Jekyll or Mr. Hyde. *J Thorac Dis* 2016; **8**: 748-750
7. Oxygen therapy and the Goldilocks principle. [editorial]. J Intensive Care Soc 2017;18(4):279.
8. Jackson RM. Pulmonary oxygen toxicity. *Chest* 1985; **88**: 900-905
9. Crapo JD. Morphologic changes in pulmonary oxygen toxicity. *Annu Rev Physiol* 1986; **48**: 721-731
10. Davis WB, Rennard SI, Bitterman PB, Crystal RG. Pulmonary oxygen toxicity. Early reversible changes in human alveolar structures induced by hyperoxia. *N Engl J Med* 1983; **309**: 878-883
11. Fandrey J, Frede S, Jelkmann W. Role of hydrogen peroxide in hypoxia-induced erythropoietin production. *Biochem J* 1994; **303**: 507-510
12. Crimi E, Sica V, Slutsky AS et al. Role of oxidative stress in experimental sepsis and multisystem organ dysfunction. *Free Radic Res* 2006; **40**: 665-672

For peer review only

For peer review only - http://bmjopen.bmj.com/site/about/guidelines.xhtml

13. Abilés J, de la Cruz AP, Castaño J et al. Oxidative stress is increased in critically ill patients according to antioxidant vitamins intake, independent of severity: a cohort study. *Crit Care* 2006; **10**: R146

14. Shyur L, Tsung J, Chen J, Chiu C, Lo C. Antioxidant properties of extracts from medicinal plants popularly used in Taiwan. *Int J Appl Sci Eng* 2005; **3**: 195-202

15. Mishra V, Baines M, Wenstone R, Shenkin A. Markers of oxidative damage, antioxidant status and clinical outcome in critically ill patients. *Ann Clin Biochem* 2005; **42**: 269-276

16. Han JE, Alvarez JA, Staitieh B et al. Oxidative stress in critically ill ventilated adults: effects of vitamin D3 and associations with alveolar macrophage function. *Eur J Clin Nutr* 2017;

17. Eastwood GM, Reade MC, Peck L, Baldwin I, Considine J, Bellomo R. Critical care nurses' opinion and self-reported practice of oxygen therapy: A survey. *Aust Crit Care* 2011; **25**: 23-30

18. Eastwood GM, Reade MC, Peck L, Jones D, Bellomo R. Intensivists' opinion and self-reported practice of oxygen therapy. *Anaesth Intensive Care* 2011; **39**: 122-126

19. Suzuki S, Eastwood G, Glassford N et al. Conservative oxygen therapy in mechanically ventilated patients: a pilot before-and- after trial. *Crit Care Med* 2014; **42**: 1414-1422

20. Helmerhorst HJ, Schultz MJ, van der Voort PH et al. Effectiveness and Clinical Outcomes of a Two-Step Implementation of Conservative Oxygenation Targets in Critically Ill Patients: A Before and After Trial. *Crit Care Med* 2016; **44**: 554-563

21. Panwar R, Hardie M, Bellomo R et al. Conservative versus Liberal Oxygenation Targets for Mechanically Ventilated Patients. A Pilot Multicenter Randomized Controlled Trial. *Am J Respir Crit Care Med* 2016; **193**: 43-51

22. Girardis M, Busani S, Damiani E et al. Effect of Conservative vs Conventional Oxygen Therapy on Mortality Among Patients in an Intensive Care Unit: The Oxygen-ICU Randomized Clinical Trial. *JAMA* 2016; **316**: 1583-1589

For peer review only - http://bmjopen.bmj.com/site/about/guidelines.xhtml

23. Ferguson ND. Oxygen in the ICU: Too Much of a Good Thing. *JAMA* 2016; **316**: 1553-1554

24. Chan AW, Tetzlaff JM, Altman DG et al. SPIRIT 2013 statement: defining standard protocol items for clinical trials. *Ann Intern Med* 2013; **158**: 200-207

For peer review only

Figure 1. Study flow diagram

For peer review only

For peer review only - http://bmjopen.bmj.com/site/about/guidelines.xhtml

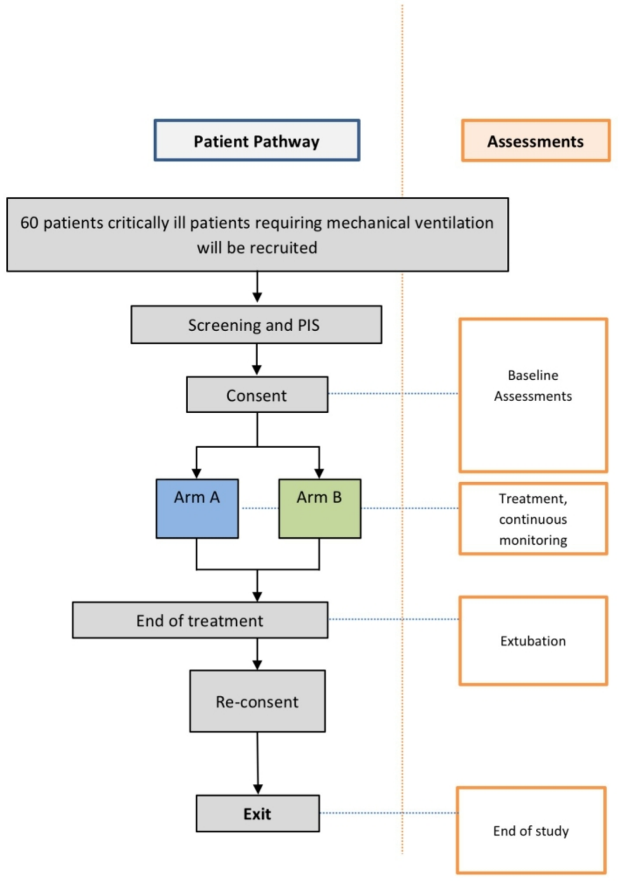

Figure 1. Study flow diagram

595x793mm (72 x 72 DPI)

TOXYC

Guidelines for patients in the **INTERVENTION** group of the **TOXYC** study: **T**argeted **OXY**gen therapy in **C**ritical illness

TARGET SpO_2 RANGE: 88 to 92%

Advice on how to maintain your patient's in the target SpO_2 range for this study:

**SpO_2** > 92%	Reduce FiO_2 in 5-10% intervals every 10 minutes until the SpO_2 is equal to or less than 92%
**SpO_2** 88-92%	Maintain SpO_2 in target range, reducing or increasing FiO_2 in 5% intervals every 10 minutes if required
**SpO_2** < 88%	Increase FiO_2 in 5-10% intervals every 10 minutes until the SpO_2 is equal to or greater than 88%

Other guidance for patients in the **INTERVENTION** group:

- Aim to use the lowest FIO_2 possible to achieve the target SpO_2.
- Try to avoid excessive use of oxygen prior to interventions such as suctioning. Increasing the FIO_2 by approximately 0.25-0.30 (25-30%) briefly should be sufficient in most stable patients.
- Once an FIO_2 of 0.21 (21%) has been reached continue to monitor SpO_2 but no further downwards titration of FIO_2 will be possible.
- Set the SpO_2 alarm limits on the monitor to: LOW = **87%**; HIGH = **93%.**
- Do not adjust the FIO_2 according to the arterial blood gas PaO_2.
- Any mode of ventilation can be used and settings such as the tidal volume, respiratory rate and PEEP can be selected by the patient's clinical team.
- Record all the patient's hourly information in the usual way.
- Please try to minimise unnecessary 100% oxygen boluses and record them all on the ICU chart.

If you have any questions or concerns about the study please contact:
daniel.martin@ucl.ac.uk *or* margaret.mcneil@nhs.net

Thank you for helping us to deliver this study.

Funded by the National Institute for Health Research and Royal Free Charity

NHS National Institute for Health Research

royal free charity

Review only

TOXYC

Guidelines for patients in the **CONTROL** group of the **TOXYC** study:
Targeted **OXY**gen therapy in **C**ritical illness

TARGET SpO_2 RANGE: ≥ 96%

Advice on how to maintain your patient's in the target SpO_2 range for this study:

**SpO_2** 96-100%	Maintain SpO_2 at or above 96% by increasing or reducing FiO_2 in 5% intervals every 10 minutes if required
**SpO_2** < 96%	Increase FiO_2 in 5-10% intervals every 10 minutes until the SpO_2 is equal to or greater than 96%

Other guidance for patients in the **CONTROL** group:

- Once an FiO_2 of 0.21 (21%) has been reached continue to monitor SpO_2 but no further downwards titration of FiO_2 will be possible.
- Set the LOW SpO_2 alarm limit on the monitor to **95%**.
- Do not adjust the FiO_2 according to the arterial blood gas PaO_2, however, if the SpO_2 is consistently at 100%, care must be taken to avoid unnecessary hyperoxaemia.
- Any mode of ventilation can be used and settings such as the tidal volume, respiratory rate and PEEP can be selected by the patient's clinical team.
- Record all the patient's hourly information in the usual way.
- Please record all 100% oxygen boluses on the ICU chart.

If you have any questions or concerns about the study please contact:
daniel.martin@ucl.ac.uk *or* margaret.mcneil@nhs.net

Thank you for helping us to deliver this study.

Funded by the National Institute for Health Research and Royal Free Charity

NHS National Institute for Health Research

royal free charity

BMJ Open

Methodology for a feasibility randomised controlled trial of targeted oxygen therapy in mechanically ventilated critically ill patients

Journal:	*BMJ Open*
Manuscript ID	bmjopen-2018-021674.R1
Article Type:	Protocol
Date Submitted by the Author:	13-Jun-2018

Complete List of Authors:	Martin, Daniel S.; University College London, Division of Surgery and Interventional Science<br>Brew-Graves, Chris; Division of Surgery and Interventional Science, University College London<br>McCartan, Neil; University College London, Surgical & Interventional Trials Unit<br>Jell, Gavin; University College London, Division of Surgery and Interventional Science<br>Potyka, Ingrid; University College London<br>Stevens, Jia; University College London, Division of Surgery and Interventional Sciences<br>Williams, Norman; University College London<br>McNeil, Margaret; Royal Free Hospital, Critical Care Unit<br>O'Driscoll, Ronan; Salford Royal NHS Foundation Trust, Respiratory medicine<br>Mythen, Monty; University College Hopital, Anaethesia<br>Grocott, Michael P. W.; University of Southampton,

**Primary Subject Heading**:	Intensive care
Secondary Subject Heading:	Respiratory medicine
Keywords:	Adult intensive & critical care < ANAESTHETICS, oxygen, hypoxia, artificial respiration, respiratory insufficiency, oxidative stress

SCHOLARONE™
Manuscripts

TITLE: Methodology for a feasibility randomised controlled trial of targeted oxygen therapy in mechanically ventilated critically ill patients

AUTHORS: * Daniel Martin[1,2], Chris Brew-Graves[3], Neil McCartan[3], Gavin Jell[2], Ingrid Potyka[3], Jia Stevens[1,2], Norman R Williams[3], Margaret McNeil[1], B Ronan O'Driscoll[4], Monty Mythen[5], Michael Grocott[6,7]

AFFILIATIONS:

1. Critical Care Unit, Royal Free Hospital, Pond Street, London, NW3 2QG
2. Division of Surgery and Interventional Science, Royal Free Campus, University College London, Royal Free Hospital, Pond Street, London, NW3 2QG
3. Surgical & Interventional Trials Unit (SITU), University College London Division of Surgery & Interventional Science, 3rd Floor, Charles Bell, House, 43 – 45 Foley Street, London, W1W 7JN
4. Manchester Academic Health Sciences Centre, and Salford Royal Foundation NHS Trust Stott Lane, Salford M6 8HD
5. Anaesthesia and Critical Care, University College London Hospitals National Institute of Health Research Biomedical Research Centre, 170 Tottenham Court Road, London, W1T 7HA
6. Integrative Physiology and Critical Illness Group, Clinical and Experimental Sciences, Faculty of Medicine, University of Southampton, Southampton, UK
7. Critical Care Research Group, Southampton NIHR Biomedical Research Centre, University Hospital Southampton, Southampton, UK

* Corresponding author: Dr Daniel Martin: Critical Care Unit, Royal Free Campus, University College London, Royal Free Hospital, Pond Street, London, NW3 2QG. +44 (0)20 7794 0500 (RFH ext 39481). daniel.martin@ucl.ac.uk

For peer review only

KEYWORDS: Oxygen; critical care; hypoxia; artificial respiration; respiratory insufficiency; oxidative stress

For peer review only

For peer review only - http://bmjopen.bmj.com/site/about/guidelines.xhtml

ABSTRACT

Introduction: Oxygen is the most commonly administered drug to mechanically ventilated critically ill adults, yet little is known about the optimum oxygen saturation (SpO_2) target for these patients; the current standard of care is an SpO_2 of 96% or above. Small pilot studies have demonstrated that permissive hypoxaemia (aiming for a lower SpO_2 than normal by using a lower fractional inspired oxygen concentration (FIO_2)) can be achieved in the critically ill and appears to be safe. This approach has not been evaluated in a National Health Service setting. It is possible that permissive hypoxaemia may be beneficial to critically ill patients thus it requires robust evaluation.

Methods and analysis: Targeted OXygen therapY in Critical illness (TOXYC) is a feasibility randomised controlled trial (RCT) to evaluate whether recruiting patients to a study of permissive hypoxaemia is possible in the UK. It will also investigate biological mechanisms that may underlie the links between oxygenation and patient outcomes. Mechanically ventilated patients with respiratory failure will be recruited from critical care units at two sites and randomised (1:1 ratio) to an SpO_2 target of either 88-92% or ≥ 96% whilst intubated with an endotracheal tube. Clinical teams can adjust FIO_2 and ventilator settings as they wish to achieve these targets. Clinical information will be collected before, during and after the intervention and blood samples taken to measure markers of systemic oxidative stress. The primary outcome of this study is feasibility, which will be assessed by recruitment rate, protocol adherence, and withdrawal rates. Secondary outcomes will include a comparison of standard critical care outcome measures between the two intervention groups, and the measurement of biomarkers of systemic oxidative stress. The results will be used to calculate a sample size, likely number of sites, and overall length of time required for a subsequent large multicentre RCT.

For peer review only - http://bmjopen.bmj.com/site/about/guidelines.xhtml

Ethics and dissemination: This study was approved by the London - Harrow Research Ethics Committee on 2nd November 2017 (REC Reference 17/LO/1334) and received HRA approval on 13th November 2017. Results from this study will be disseminated in peer reviewed journals, the NIHR Journals Library and patient information websites.

The study has been funded by the National Institute of Health Research and Royal Free Charity. It is registered on www.clinical trial.gov (NCT03287466).

For peer review only

STRENGTHS AND LIMITATIONS OF THIS STUDY

- This is the first study of permissive hypoxaemia in critically ill patients in a National Health Service setting
- It is a small randomised controlled trial to assess feasibility, not the efficacy of the intervention
- The study will compare levels of biomarkers of systemic oxidative stress between the two intervention groups
- It will provide valuable information to enable the design of future large-scale randomised controlled trials

For peer review only - http://bmjopen.bmj.com/site/about/guidelines.xhtml

INTRODUCTION

In the UK there are over 190,000 admissions to adult critical care units each year (www.icnarc.org). Approximately 40% of these patients will require mechanical ventilation and the mortality rate in this group is approximately 30%.[1, 2] Hypoxaemia is common amongst this cohort of patients and we lack evidence-based guidelines for their management, particularly regarding what levels of arterial oxygenation are acceptable or optimal. It has been proposed that attempting to fully reverse hypoxaemia in critically ill patients may pose a greater risk of harm than allowing moderate hypoxaemia to persist, a concept called permissive hypoxaemia.[3] The premise behind permissive hypoxaemia is that the interventions used to correct hypoxaemia may themselves cause harm, in particular high concentrations of inspired oxygen, therefore safely minimising their use could be beneficial.[4–7]

Oxygen has the potential to cause harm when used in high concentration, primarily via its toxic effect on the lungs.[8–10] Reactive oxygen species (ROS) released mainly from the inner mitochondrial membrane during oxidative phosphorylation serve an essential role in cellular signalling but in excess these highly reactive molecules are able to destroy lipids, proteins and DNA. Their rate of release is determined by cellular oxygen tension[11] and the extent of damage caused by them can be measured by evaluating biomarkers of tissue degradation[12]. The lung parenchyma is particularly susceptible to oxygen toxicity in critically ill patients as a result of being exposed to high concentration oxygen during mechanical ventilation. The threshold above which harm may be caused (in terms of concentration and duration of exposure) in critically ill patients is unclear, but since lung injury is common in this patient cohort the threshold may well be lower than in other patients or healthy volunteers. During critical illness the propagation of pro-inflammatory pathways, with the activation of leukocyte and vascular endothelial responses further increase the ROS burden.[13] This depletes endogenous antioxidants, which normally

For peer review only

For peer review only - http://bmjopen.bmj.com/site/about/guidelines.xhtml

regulate ROS homeostasis.[14] As a consequence, oxidative stress is a key mechanism of injury in systemic multi-organ failure, and has been linked to increase in morbidity and mortality in critical illness. [15, 16]

Oxygen is a drug a relatively narrow therapeutic index. It should therefore be prescribed, administered and monitored in a manner comparable to other drugs that have toxic side effects. There appears, however, to be wide variation in practice regarding its use and opinions about oxygenation in the critically ill. [17, 18] This is perhaps the result of a paucity of evidence from robust clinical trials; a somewhat surprising situation given that almost every patient admitted to a critical care unit will receive supplementary oxygen. The traditional teaching that hypoxaemia must be avoided at all costs, may have led to a disregard to the potential harm caused by excessive oxygen, and this requires evaluation.

A small number of studies have begun to explore permissive hypoxaemia as a viable treatment strategy in the critically ill, primarily assessing feasibility and safety. In the first study of its kind, 105 mechanically ventilated patients were assessed in a before (n=51) and after (n=54) design. [19] Following a period of standard oxygenation practice in a single centre (aiming for normal or high blood oxygen levels), a practice change was initiated in which oxygen saturation (SpO_2) was maintained at 90–92%. The authors of this study concluded that the conservative oxygen therapy intervention was feasible and free of adverse biochemical, physiological, or clinical outcomes. A comparable strategy was used on a much larger scale in a two-stage model, moving from normal oxygenation to an SpO_2 of 92–95%.[20] These authors reported that mechanical ventilation time was significantly lower during both study (lower SpO_2) phases compared to baseline. The adjusted ICU mortality and ICU-free days did not significantly differ between study phases but mortality decreased in reference to baseline for both of the low SpO_2 phases. In the first multicentre randomised controlled trial (RCT) of permissive hypoxaemia a

For peer review only - http://bmjopen.bmj.com/site/about/guidelines.xhtml

total of 103 mechanically ventilated patients were allocated to either a conservative oxygenation group (SpO_2 of 88–92%) or a liberal oxygenation group (SpO_2 of greater than or equal to 96%.[21] The purpose of the study was to confirm feasibility and this was demonstrated, along with excess of adverse events in the low SpO_2 group. The most recently published trial of permissive hypoxaemia was a single centre RCT that compared SpO_2 targets of 94%-98% versus 97-100%.[22] The primary outcome of this study was ICU mortality, and the values reported were: 11.6% in the conservative group and 20.2% in the conventional group, giving an absolute risk reduction of 8.6% (1.7-15.0%). This study had a number of limitations[23] but still adds weight to the argument that permissive hypoxaemia appears to not be harmful and may be of benefit.

A factor of great importance to the design of future studies is selecting the correct 'standard' treatment group, in order that the comparison to an intervention of lower oxygenation is valid and meaningful. Different studies have approached this in different ways (either by aiming for an oxygenation target or by determining the administered concentration of oxygen). We hope this feasibility will evaluate our selected methodology and allow us to compare it to other approaches that have been used. We also hope the results of this study will allow us to understand more about other issues specific to critically ill patients such as the concomitant presence of anaemia, low cardiac output and acute respiratory distress syndrome and chronic obstructive pulmonary disease.

METHODS

The trial was designed according to the SPIRIT (Standard Protocol Items: Recommendations for Interventional Trials) statements.[24]

For peer review only - http://bmjopen.bmj.com/site/about/guidelines.xhtml

Trial aim and objectives

The Targeted OXYgen therapy in Critical illness (TOXYC) study aims to determine whether reducing the SpO_2 target in patients requiring mechanical ventilation is feasible (in terms of participant recruitment and delivery of the intervention) in a National Health Service (NHS) setting. In doing this we hope to inform future investigators who wish to construct larger trials in this field. The objectives are to construct a randomised controlled trial of conventional oxygenation versus permissive hypoxaemia, identify any potential barriers to research in this field and explore biological mechanisms that may explain the proposed benefits from the intervention. The project was favourably supported at the UK Critical Care Research Forum in 2016.

Primary outcome measure

The primary outcome of this study is feasibility, which will be assessed in the following ways: i) the ability to recruit patients (recruitment rate), ii) protocol deviations, iii) rate of withdrawal from the study (in both the intervention and control groups), and iv) the reasons for any withdrawal from the study.

Feasibility of recruitment will be evaluated by monitoring patient screening and subsequent agreement to participate, along with any withdrawal of consent during or after the study. Implementation of the study protocol will be evaluated by analysing adherence to oxygenation targets and completion of the treatment without protocol deviations. Reasons for withdrawal will be assessed by the trial management group at the end of the study to assess whether there are common themes that can be addressed in the future.

Secondary outcome measures

Measurements of oxidative stress (including 4- hydroxynonenal, protein carbonyls, total antioxidant capacity and glutathione reductase) will be made in blood samples taken from

For peer review only - http://bmjopen.bmj.com/site/about/guidelines.xhtml
For peer review only

participants to understand the potential biological mechanisms that link blood oxygen levels to clinical outcomes. In addition, routine clinical data and outcome measures will be collected from the participants to assess any adverse effects caused by the intervention. Finally, length of critical care stay, length of hospital stay, and survival at critical care unit discharge, 30 and 90 days will be collected. This information will be essential for the design of future larger trials.

Trial design

TOXYC, a multi-centre RCT, which will be conducted at two sites, is a trial of targeted oxygen therapy in adult critically ill patients receiving mechanical ventilation via an endotracheal tube. Sixty patients will be allocated on a 1:1 basis to either a normal SpO_2 target group or a lower than normal SpO_2 target group. A flow diagram of the study is shown in Figure 1. Recruitment began in February 2018 and is planned to continue for 15 months.

Selection of participants

Screening

Screening will occur as part of routine research activity on the two critical care units involved in the study. Research nurses will use medical notes to determine initial suitability for the study, according to the inclusion and exclusion criteria. No additional tests or examinations will be required to ascertain whether patients are eligible for the study. Screening will occur as patients are admitted to the critical care units to minimise the time from admission to enrolment.

Inclusion criteria

- Unplanned admission to a critical care unit
- 18 years of age and above (no upper age limit)
- Respiratory failure forms part of the admission diagnosis
- Enrolled within 24 hours of admission (if already intubated) or within 24 hours of intubation (if intubated on ICU)

For peer review only

- The patient is expected to receive mechanical ventilation for > 72 hours

Exclusion criteria

- Admission following surgery (elective or unplanned)
- Those patients expected to die within 24 hours of admission to ICU
- Pregnant females
- Admission post-cardiac arrest
- Patients with chronic lung disease known (or highly suspected) to have baseline oxygen saturations in the range of the intervention arm (i.e. 88-92%)
- Admission post trauma (including traumatic brain injury)
- Known sickle cell trait or disease
- Ongoing significant haemorrhage or profound anaemia
- Severe peripheral vascular disease
- Severe pulmonary hypertension
- Other medical conditions where mild hypoxaemia would be contra-indicated
- Patients participating in other interventional clinical trials

For peer review only

Enrolment

Consent

Due to the severity of illness of the patients being recruited to this study, and the use of sedative drugs that are required for mechanical ventilation, it is unlikely that potential participants will have capacity. Should a potential participant be deemed to have capacity they will be approached by the research team, given a patient information sheet (PIS) and then provided with an opportunity to ask questions. After an appropriate length of time, the research team will seek informed consent from the patient if they wish to participate.

For peer review only - http://bmjopen.bmj.com/site/about/guidelines.xhtml

If the patient lacks capacity to provide informed consent, a Personal Consultee (PeC) will be appointed to represent them. This could be the patient's next of kin, a relative or close friend with whom to discuss the patient's participation in the trial. The research team will seek the PeC's opinion as to whether patient would wish to take part in the trial, providing for them an appropriate version of the ethically approved PIS. If the PeC believes the patient would have wanted to participate in the study (or would not have objected to it), they will be asked to sign a PeC agreement form. If there is no PeC present or immediately available in person, opinion may be sought from a suitable person via the telephone and then a telephone agreement form completed by a member of the research team.

If there is no identifiable PeC for a potential participant then they will be provided with a Professional Consultee (PrC), who is completely independent of the study. Their opinion will be sought as to whether it is appropriate for the patient to be enrolled into the study. Opinion will be sought in the same manner as for the PeC, involving the appropriate version of the ethically approved PIS.

Adequate time will be given for consideration by the patient or PeC/PrC to consider the information in the PIS and ask questions. The research team will record when the PIS has been given to the patient or their consultee. Due to the nature of this patient cohort (critically ill patients requiring substantial organ support due to the severity of their illness) the length of time from identifying a potential participant to initiating the intervention is likely to be less than 24 hours. This is to avoid dilution of the intervention or control effect prior to its commencement.

If a participant who lacked capacity at the point of recruitment subsequently becomes able to provide informed consent (because they gain capacity on recovery from their illness), they will be informed about their participation in the study, provided with a PIS and asked whether they would be willing to provide retrospective consent. At this point the participant will be given the

For peer review only - http://bmjopen.bmj.com/site/about/guidelines.xhtml

opportunity to withdraw from the study and to decide if the data (and blood samples) collected from them can be included in the final analysis.

All patient consent and consultee agreement procedures will adhere to the Mental Capacity Act (2005).

Randomisation

Randomisation will be carried out online after a patient has been recruited to the study and an agreement form or consent form has been signed. It will be conducted in a 1:1 manner for the intervention and control groups, stratified by study site, using random permuted blocks of different size. The process of randomisation will be conducted online (www.sealedenvelope.com).

Withdrawal

Participants will be withdrawn from the study if:

- The responsible clinician deems it inappropriate for treatment to continue due to a change in the patient's condition
- The Chief Investigator or delegated member of the research team deems it inappropriate for treatment to continue due to a change in the patient's condition
- Agreement for the patient to participate in the study is withdrawn by the PeC or PrC
- The patient regains capacity and choses to withdraw from the study

As this is a feasibility study, patients withdrawn from the study will not be replaced, but the reason for withdrawal will be recorded.

For peer review only - http://bmjopen.bmj.com/site/about/guidelines.xhtml

Trial treatment

Intervention

The intervention is a more conservative use of oxygen via the ventilator to achieve an SpO_2 of 88-92%, lower than normal practice in most critical care units in the UK. The intervention will be delivered by the participant's clinical team, which will consist of the critical care doctors and nurses at the two study centres. These teams will be provided with guidance to help keep participants within their target SpO_2 (*supplementary material 1*) but this will not be protocolised.

Comparator

The control group will receive oxygen to maintain an SpO_2 at 96% or above (standard care). As per the intervention group, guidance will be provided to the clinical team to help maintain participants within their target SpO_2 (*supplementary material 2*).

Duration of treatment

The aim is for the intervention to be commenced as soon as possible after admission to the critical care unit (following enrolment) and end following removal of the participant's endotracheal tube. Specific treatment end-points for both groups would therefore include i) extubation, ii) formation of a tracheostomy, iii) transfer to another critical care unit, and iv) death. The research team will review enrolled participants daily to monitor adherence to SpO_2 targets and provide bedside advice where required. No targets or limitations will be set for arterial partial pressure of oxygen (PaO_2) or carbon dioxide ($PaCO_2$). Should a patient in the study be transferred out of the ICU for a short period of time (e.g. for an investigation or intervention) the protocol will be paused until their return. Whilst out of the ICU the clinical team will be in control of the patient's oxygenation. Should a patient's condition deteriorate to such an extent that either the clinical or research team feel it not in the patient's best interest to continue in the study, they will be withdrawn from it at that point.

For peer review only - http://bmjopen.bmj.com/site/about/guidelines.xhtml

For peer review only

Standard clinical management.

Aside from the designated SpO_2 targets, all other aspects of care will remain the same between the intervention and control groups. Regular arterial blood gases should be taken during the trial period, according to local clinical guidelines; no additional arterial blood gases will be necessary for the purpose of the study.

Data collection

Data will be collected from various sources including the participant's medical records, bedside charts and hospital computer systems. Data will be collected from these primary sources by members of the research team and entered into an electronic clinical record form (eCRF).

Baseline data collection

- Patient demographics: age, gender, height and weight
- Cause of respiratory failure (diagnosis)
- The presence of any chronic diseases
- Acute Physiology and Chronic Health Evaluation (APACHE) II score (and its components)
- Sequential Organ Failure Assessment (SOFA) score (and its components)
- *Respiratory measurements*: PaO_2, $PaCO_2$, pH, SpO_2, FIO_2, ventilator settings and measures
- *Cardiovascular measurements*: blood pressure, heart rate, cardiac rhythm, vasopressor / inotrope dose, fluid balance
- *Renal measurements*: creatinine, urine output in past 24 hours, the need for renal replacement therapy
- *Hepatic measurements*: transaminases, blood clotting values and bilirubin
- Blood lactate concentration

For peer review only - http://bmjopen.bmj.com/site/about/guidelines.xhtml

Subsequent data collection during treatment

- Most measures will be taken daily, except for those specifically related to oxygenation, which will be collected hourly, to permit detailed analysis of compliance to blood oxygenation target.
- Time to extubation or detachment from mechanical ventilation, and mechanical ventilation free days on ICU
- Adverse events occurring during the study period

Follow up

- Length of ICU stay
- Length of hospital stay
- 30 and 90 day survival rates, and days alive out of hospital
- Adverse events

Data Management

This trial will use an eCRF and trial data will be entered into an approved, protected database (https://www.elsevier.com/solutions/macro). Access to the eCRF system will only be provided to staff with relevant authority. Participants will be given a unique subject number and subject identifier. Data will be entered under this identification number onto the central database stored on the servers. The database will be password protected and only accessible to members of the TOXYC study team and external regulators if requested. At site, access will only be granted to staff with permission on the delegation log, and after training. The servers are protected by firewalls and are patched and maintained according to best practice. The physical location of the servers is protected by CCTV and security door access. The database software provides a number of features to help maintain data quality, including; maintaining an audit trail, allowing custom validations on all data, allowing users to raise data query requests, and search facilities

For peer review only

to identify validation failure/ missing data.

The identification, screening and enrolment logs, linking participant identifiable data to the pseudo-anonymised subject numbers will either be held in written form in a locked filing cabinet or electronically in password protected form on hospital computers. After completion of the study the identification, screening and enrolment logs will be stored securely by the sites for 20 years. After completion of the study the identification, screening and enrolment logs will be stored securely by the sites for 20 years.

Sample collection, storage and processing

Blood samples will be taken from participants in order to evaluate oxidative stress. Samples will be taken from an indwelling arterial catheter that is already present in the patient as part of routine critical care. Blood will be processed at each of the two centres according to a defined standard operating procedure, and then stored at -80 degrees centigrade. These blood samples will be taken at baseline (shortly after the patient has been recruited into the study but prior to their treatment being commenced), and on days 2, 3, 5 and 10 after recruitment. A number of biomarkers of oxidative stress will be measured, including: 4- hydroxyl-2-nonenal, protein carbonyls, total antioxidant capacity and glutathione reductase.

Safety monitoring

All adverse events will be recorded in the medical records and reported to the appropriate body; either online to SITU or by email to the sponsor. Table 1 shows a basic list of expected adverse events that will be recorded within the patient’s eCRF and medical notes, but the sponsor will not be informed. Adverse events (AEs) and serious adverse events (SAEs) will be summarised descriptively by interventional group at the end of the study.

For peer review only - http://bmjopen.bmj.com/site/about/guidelines.xhtml

RESPIRATORY	CARDIOVASCULAR	HAEMATOLOGICAL
• reintubation<br>• arterial desaturation<br>• pneumothorax<br>• pleural effusion<br>• pneumonia<br>• pulmonary embolism	• arrhythmia<br>• hypotension<br>• requirement for inotropic support	• Anaemia<br>• Low platelet count<br>• High white blood cell count
**RENAL**	**GASTROINTESTINAL**	**NEUROLOGICAL**
• Acute kidney injury<br>• Requirement for renal support<br>• hyperkalaemia	• Diarrhoea<br>• vomiting<br>• Failure to absorb enteral feed	• Delirium / agitation

Table 1. A list of expected adverse events that may occur during the course of the study

For peer review only

Trial monitoring and oversight

The TOXYC trial will report to a data monitoring committee, and a trial steering committee will be appointed to provide study oversight on behalf of the sponsor and funder. Day to day management of the trial will be the responsibility of the trial coordinator with oversight from the trial management group.

Statistics

No formal comparative analyses are planned in this feasibility study. The primary and secondary outcome measures will be presented using summary statistics (e.g. means, standard deviations, medians, proportions). Missing data, non-compliers and withdrawals will be looked at in detail to determine if there is any evidence of bias.

A CONSORT diagram will be completed, summarising the number of patients eligible for the

For peer review only - http://bmjopen.bmj.com/site/about/guidelines.xhtml

study, the number randomised in each arm, and enumerating in detail those not approached (with reasons), the number of withdrawals (with reasons) overall and per arm. Recruitment rate (overall, per site, and peak recruitment rate per month) will be determined. Monthly and cumulative accrual graphs will be constructed. Baseline characteristics of randomised participants will be summarised, including gender, age, height, weight, details of medical history, pre-intervention APACHE II and SOFA scores. Mean and SD, or median and IQR, will be calculated as appropriate. For the secondary objectives, summary statistics on respiratory, cardiovascular, renal, and hepatic measurements will be calculated at the appropriate time points (hourly or daily). Length of stay in the critical care unit and in hospital will be summarised. 30 and 90 day mortality rates will be calculated, and "days alive and out-of-hospital" determined for each patient and summarised using appropriate measures. Compliance will be assessed. For each patient, the proportion of time spent within the randomisation-determined oxygen saturation limits will be calculated, and summarised by treatment arm. Adverse events will be tabulated and grouped according to seriousness, severity and causality.

All variables will be checked for completeness and checked for the presence of outliers. Graphical depictions of results will be prepared, both on a per-patient basis (especially for the longitudinal data such as hourly oxygen measurement) and grouped by intervention. Frequency distribution curves will be shown where appropriate (especially for the "length of stay" measurements).

No hypothesis testing is envisaged for this feasibility study. The results will be used to calculate a sample size, likely number of sites, and overall length of time required for a subsequent large multicentre RCT.

For peer review only - http://bmjopen.bmj.com/site/about/guidelines.xhtml

Ethical compliance

This Trial was approved by the London - Harrow Research Ethics Committee on 02NOV17 (REC Reference 17/LO/1334) and received HRA approval on 13NOV17. TOXYC is also registered on www.clinical trial.gov (NCT03287466)

Patient and public involvement

In 2014, the UK Intensive Care Society published the results of their James Lind Alliance (JLA) Priority Setting Partnership [25]. The aim was to identify and prioritise unanswered questions about adult critical care that are important to people who have been critically ill, their families, and the health professionals who care for them. One of the identified priorities for research was: *"What is the best way of preventing damage to the lungs of patients receiving respiratory support (ventilation)?"* This study addresses this publicly driven need, by assessing a treatment strategy that has the potential to reduce iatrogenic lung injury to patients on a ventilator and therefore improve survival. At the Royal Free Hospital critical care unit we have a growing group of patients and relatives who are willing to assist with the development of research. This group was formed and is managed by our research team at the Royal Free Hospital. A volunteer member of the public from this group agreed to assist us throughout the study, from the application for funding to dissemination of the findings. This person was a co-applicant on the grant application and is an invited member of the Trial Steering Committee. We hope that including a member of the public in the design and delivery of the study will improve the experience of participants in this an future clinical research projects.

CONCLUSION

The results of this feasibility trial will inform researchers about the ability to conduct a study evaluating permissive hypoxaemia in critically ill patients. It will provide information about

For peer review only - http://bmjopen.bmj.com/site/about/guidelines.xhtml

recruitment rates in UK critical care units and help to identify any barriers to future research. Furthermore, results from oxidative stress marker analysis may highlight biological markers of importance in the pathway between oxygen administration and patient outcomes.

Funding statement: This work was supported by the National Institute for Health Research (PB-PG-0815-20006) and Royal Free Charity.

For peer review only

Authors' contributions:

DM: Conceived idea, designed study, obtained funding for RCT, obtained funding for mechanistic component, obtained ethics approval, will be CI for study and PI at Royal Free, wrote manuscript, revised manuscript

CB-G: obtained ethics approval, responsible for trial governance, wrote manuscript, revised manuscript

NM: obtained ethics approval, responsible for trial governance, revised manuscript

GJ: obtained funding for mechanistic component, lead for biochemical analysis, revised manuscript

IP: responsible for data collection, revised manuscript

JS: involved in sample processing, revised manuscript

NW: obtained ethics approval, responsible for trial governance, responsible for data analysis and statistics, wrote manuscript, revised manuscript

MMcN: lead research nurse for study, responsible for screening patients and collecting data, revised manuscript

BRO'D: obtained funding for RCT, involved in study design, revised manuscript

MM: Conceived idea, designed study, obtained funding for RCT, revised manuscript

For peer review only - http://bmjopen.bmj.com/site/about/guidelines.xhtml

MG: Conceived idea, designed study, obtained funding for RCT, PI at Southampton General Hospital, wrote manuscript, revised manuscript

Competing interests statement:

DM, MM and MG are directors of a company developing an oxygen delivery device (Oxygen Control Ltd).

DM has received honoraria and consultancy fees from Siemens Healthcare, Masimo, Deltex and Edwards Lifesciences.

MG is the National Specialty Lead for Anaesthesia, Perioperative Medicine and Pain within the UK National Institute of Heath Research Clinical Research Network, an elected council member of the Royal College of Anaesthetists and serves on the board of the Evidence Based Perioperative Medicine (EBPOM) social enterprise and the medical advisory board of Sphere Medical Ltd. MG has received honoraria for speaking and/or travel expenses from Edwards Lifesciences, Fresenius-Kabi, BOC Medical (Linde Group), Ely-Lilly Critical Care, and Cortex GmBH. MG is executive chair of the Xtreme-Everest Oxygen Research Consortium and joint Editor-in-Chief of the journal *Perioperative Medicine.*

MM is a consultant for Baxter, Edwards Lifesciences and Deltex; his University Chair is supported by Smiths Medical; Elected Council Member Royal College of Anaesthetsists; Editorial Board BJA and Critical Care; Founding Editor-in-Chief Perioperative Medicine.

JS, NW, ROD, GJ and MMcN have no competing interests.

This article presents independent research funded by the National Institute for Health Research (NIHR) under its Research for Patient Benefit (RfPB) Programme (Grant Reference Number PB-PG-0815-20006). The views expressed are those of the authors and not necessarily those of the NHS, the NIHR or the Department of Health.

For peer review only

For peer review only - http://bmjopen.bmj.com/site/about/guidelines.xhtml

For peer review only

For peer review only - http://bmjopen.bmj.com/site/about/guidelines.xhtml

REFERENCES

1. Eastwood G, Bellomo R, Bailey M et al. Arterial oxygen tension and mortality in mechanically ventilated patients. *Intensive Care Med*. 2012;38:91-98.
2. de Jonge E, Peelen L, Keijzers PJ et al. Association between administered oxygen, arterial partial oxygen pressure and mortality in mechanically ventilated intensive care unit patients. *Crit Care*. 2008;12:R156.
3. Martin DS, Grocott MP. Oxygen therapy in critical illness: precise control of arterial oxygenation and permissive hypoxemia. *Crit Care Med*. 2013;41:423-432.
4. Abdelsalam M, Cheifetz IM. Goal-directed therapy for severely hypoxic patients with acute respiratory distress syndrome: permissive hypoxemia. *Respir Care*. 2010;55:1483-1490.
5. Capellier G, Panwar R. Is it time for permissive hypoxaemia in the intensive care unit? *Crit Care Resusc*. 2011;13:139-141.
6. He HW, Liu DW. Permissive hypoxemia/conservative oxygenation strategy: Dr. Jekyll or Mr. Hyde. *J Thorac Dis*. 2016;8:748-750.
7. Oxygen therapy and the Goldilocks principle. [editorial]. J Intensive Care Soc 2017;18(4):279.
8. Jackson RM. Pulmonary oxygen toxicity. *Chest*. 1985;88:900-905.
9. Crapo JD. Morphologic changes in pulmonary oxygen toxicity. *Annu Rev Physiol*. 1986;48:721-731.
10. Davis WB, Rennard SI, Bitterman PB, Crystal RG. Pulmonary oxygen toxicity. Early reversible changes in human alveolar structures induced by hyperoxia. *N Engl J Med*. 1983;309:878-883.

For peer review only - http://bmjopen.bmj.com/site/about/guidelines.xhtml

11. Fandrey J, Frede S, Jelkmann W. Role of hydrogen peroxide in hypoxia-induced erythropoietin production. *Biochem J*. 1994;303:507-510.

12. Crimi E, Sica V, Slutsky AS et al. Role of oxidative stress in experimental sepsis and multisystem organ dysfunction. *Free Radic Res*. 2006;40:665-672.

13. Abilés J, de la Cruz AP, Castaño J et al. Oxidative stress is increased in critically ill patients according to antioxidant vitamins intake, independent of severity: a cohort study. *Crit Care*. 2006;10:R146.

14. Shyur L, Tsung J, Chen J, Chiu C, Lo C. Antioxidant properties of extracts from medicinal plants popularly used in Taiwan. *Int J Appl Sci Eng*. 2005;3:195-202.

15. Mishra V, Baines M, Wenstone R, Shenkin A. Markers of oxidative damage, antioxidant status and clinical outcome in critically ill patients. *Ann Clin Biochem*. 2005;42:269-276.

16. Han JE, Alvarez JA, Staitieh B et al. Oxidative stress in critically ill ventilated adults: effects of vitamin D3 and associations with alveolar macrophage function. *Eur J Clin Nutr*. 2017

17. Eastwood GM, Reade MC, Peck L, Baldwin I, Considine J, Bellomo R. Critical care nurses' opinion and self-reported practice of oxygen therapy: A survey. *Aust Crit Care*. 2011;25:23-30.

18. Eastwood GM, Reade MC, Peck L, Jones D, Bellomo R. Intensivists' opinion and self-reported practice of oxygen therapy. *Anaesth Intensive Care*. 2011;39:122-126.

For peer review only - http://bmjopen.bmj.com/site/about/guidelines.xhtml

19. Suzuki S, Eastwood G, Glassford N et al. Conservative oxygen therapy in mechanically ventilated patients: a pilot before-and- after trial. *Crit Care Med*. 2014;42:1414-1422.

20. Helmerhorst HJ, Schultz MJ, van der Voort PH et al. Effectiveness and Clinical Outcomes of a Two-Step Implementation of Conservative Oxygenation Targets in Critically Ill Patients: A Before and After Trial. *Crit Care Med*. 2016;44:554-563.

21. Panwar R, Hardie M, Bellomo R et al. Conservative versus Liberal Oxygenation Targets for Mechanically Ventilated Patients. A Pilot Multicenter Randomized Controlled Trial. *Am J Respir Crit Care Med*. 2016;193:43-51.

22. Girardis M, Busani S, Damiani E et al. Effect of Conservative vs Conventional Oxygen Therapy on Mortality Among Patients in an Intensive Care Unit: The Oxygen-ICU Randomized Clinical Trial. *JAMA*. 2016;316:1583-1589.

23. Ferguson ND. Oxygen in the ICU: Too Much of a Good Thing. *JAMA*. 2016;316:1553-1554.

24. Chan AW, Tetzlaff JM, Altman DG et al. SPIRIT 2013 statement: defining standard protocol items for clinical trials. *Ann Intern Med*. 2013;158:200-207.

25. Reay H, Arulkumaran N, Brett S. Priorities for future intensive care research in the UK: results of a James Lind alliance priority setting partnership. *Journal of the Intensive Care Society*. 2014;15.4:288-296.

For peer review only

For peer review only - http://bmjopen.bmj.com/site/about/guidelines.xhtml

Figure 1. Study flow diagram

For peer review only

For peer review only - http://bmjopen.bmj.com/site/about/guidelines.xhtml

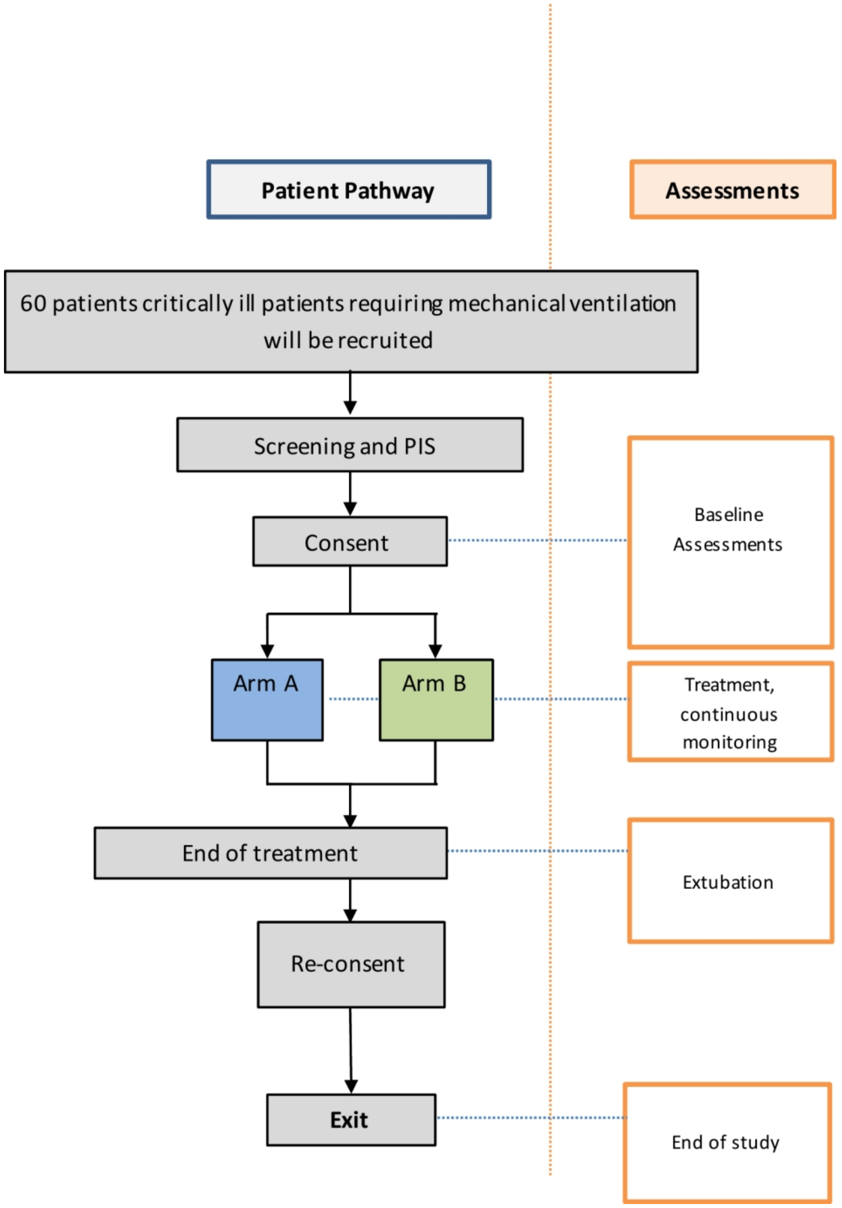

Figure 1. Study flow diagram

127x184mm (300 x 300 DPI)

TOXYC

Guidelines for patients in the **INTERVENTION** group of the **TOXYC** study: **T**argeted **OXY**gen therapy in **C**ritical illness

TARGET SpO_2 RANGE: 88 to 92%

Advice on how to maintain your patient's in the target SpO_2 range for this study:

**SpO_2** > 92%	Reduce FIO_2 in 5-10% intervals every 10 minutes until the SpO_2 is equal to or less than 92%
**SpO_2** 88-92%	Maintain SpO_2 in target range, reducing or increasing FIO_2 in 5% intervals every 10 minutes if required
**SpO_2** < 88%	Increase FIO_2 in 5-10% intervals every 10 minutes until the SpO_2 is equal to or greater than 88%

Other guidance for patients in the **INTERVENTION** group:

- Aim to use the lowest FIO_2 possible to achieve the target SpO_2.
- Try to avoid excessive use of oxygen prior to interventions such as suctioning. Increasing the FIO_2 by approximately 0.25-0.30 (25-30%) briefly should be sufficient in most stable patients.
- Once an FIO_2 of 0.21 (21%) has been reached continue to monitor SpO_2 but no further downwards titration of FIO_2 will be possible.
- Set the SpO_2 alarm limits on the monitor to: LOW = **87%**; HIGH = **93%.**
- Do not adjust the FIO_2 according to the arterial blood gas PaO_2.
- Any mode of ventilation can be used and settings such as the tidal volume, respiratory rate and PEEP can be selected by the patient's clinical team.
- Record all the patient's hourly information in the usual way.
- Please try to minimise unnecessary 100% oxygen boluses and record them all on the ICU chart.

If you have any questions or concerns about the study please contact:
daniel.martin@ucl.ac.uk *or* margaret.mcneil@nhs.net

Thank you for helping us to deliver this study.

Funded by the National Institute for Health Research and Royal Free Charity

NHS National Institute for Health Research

royal free charity

TOXYC

Guidelines for patients in the **CONTROL** group of the **TOXYC** study: **T**argeted **OXY**gen therapy in **C**ritical illness

TARGET SpO_2 RANGE: ≥ 96%

Advice on how to maintain your patient's in the target SpO_2 range for this study:

**SpO_2** 96-100%	Maintain SpO_2 at or above 96% by increasing or reducing FiO_2 in 5% intervals every 10 minutes if required
**SpO_2** < 96%	Increase FiO_2 in 5-10% intervals every 10 minutes until the SpO_2 is equal to or greater than 96%

Other guidance for patients in the **CONTROL** group:

- Once an FiO_2 of 0.21 (21%) has been reached continue to monitor SpO_2 but no further downwards titration of FiO_2 will be possible.
- Set the LOW SpO_2 alarm limit on the monitor to **95%**.
- Do not adjust the FiO_2 according to the arterial blood gas PaO_2, however, if the SpO_2 is consistently at 100%, care must be taken to avoid unnecessary hyperoxaemia.
- Any mode of ventilation can be used and settings such as the tidal volume, respiratory rate and PEEP can be selected by the patient's clinical team.
- Record all the patient's hourly information in the usual way.
- Please record all 100% oxygen boluses on the ICU chart.

If you have any questions or concerns about the study please contact:
daniel.martin@ucl.ac.uk *or* margaret.mcneil@nhs.net

Thank you for helping us to deliver this study.

Funded by the National Institute for Health Research and Royal Free Charity

NHS National Institute for Health Research

royal free charity

BMJ Open

Protocol for a feasibility randomised controlled trial of targeted oxygen therapy in mechanically ventilated critically ill patients

Journal:	*BMJ Open*
Manuscript ID	bmjopen-2018-021674.R2
Article Type:	Protocol
Date Submitted by the Author:	10-Oct-2018
Complete List of Authors:	Martin, Daniel S.; University College London, Division of Surgery and Interventional Science<br>Brew-Graves, Chris; Division of Surgery and Interventional Science, University College London<br>McCartan, Neil; University College London, Surgical & Interventional Trials Unit<br>Jell, Gavin; University College London, Division of Surgery and Interventional Science<br>Potyka, Ingrid; University College London<br>Stevens, Jia; University College London, Division of Surgery and Interventional Sciences<br>Williams, Norman; University College London<br>McNeil, Margaret; Royal Free Hospital, Critical Care Unit<br>O'Driscoll, Ronan; Salford Royal NHS Foundation Trust, Respiratory medicine<br>Mythen, Monty; University College Hopital, Anaethesia<br>Grocott, Michael P. W.; University of Southampton,
**Primary Subject Heading**:	Intensive care
Secondary Subject Heading:	Respiratory medicine
Keywords:	Adult intensive & critical care < ANAESTHETICS, oxygen, hypoxia, artificial respiration, respiratory insufficiency, oxidative stress

SCHOLARONE™
Manuscripts

TITLE: Protocol for a feasibility randomised controlled trial of targeted oxygen therapy in mechanically ventilated critically ill patients

AUTHORS: * Daniel Martin[1,2], Chris Brew-Graves[3], Neil McCartan[3], Gavin Jell[2], Ingrid Potyka[3], Jia Stevens[1,2], Norman R Williams[3], Margaret McNeil[1], B Ronan O'Driscoll[4], Monty Mythen[5], Michael Grocott[6,7]

AFFILIATIONS:

1. Critical Care Unit, Royal Free Hospital, Pond Street, London, NW3 2QG

2. Division of Surgery and Interventional Science, Royal Free Campus, University College London, Royal Free Hospital, Pond Street, London, NW3 2QG

3. Surgical & Interventional Trials Unit (SITU), University College London Division of Surgery & Interventional Science, 3rd Floor, Charles Bell, House, 43 – 45 Foley Street, London, W1W 7JN

4. Manchester Academic Health Sciences Centre, and Salford Royal Foundation NHS Trust Stott Lane, Salford M6 8HD

5. Anaesthesia and Critical Care, University College London Hospitals National Institute of Health Research Biomedical Research Centre, 170 Tottenham Court Road, London, W1T 7HA

6. Integrative Physiology and Critical Illness Group, Clinical and Experimental Sciences, Faculty of Medicine, University of Southampton, Southampton, UK

7. Critical Care Research Group, Southampton NIHR Biomedical Research Centre, University Hospital Southampton, Southampton, UK

* Corresponding author: Dr Daniel Martin: Critical Care Unit, Royal Free Campus, University College London, Royal Free Hospital, Pond Street, London, NW3 2QG. +44 (0)20 7794 0500 (RFH ext 39481). daniel.martin@ucl.ac.uk

For peer review only

For peer review only - http://bmjopen.bmj.com/site/about/guidelines.xhtml

KEYWORDS: Oxygen; critical care; hypoxia; artificial respiration; respiratory insufficiency; oxidative stress

For peer review only

For peer review only - http://bmjopen.bmj.com/site/about/guidelines.xhtml

ABSTRACT

Introduction: Oxygen is the most commonly administered drug to mechanically ventilated critically ill adults, yet little is known about the optimum oxygen saturation (SpO_2) target for these patients; the current standard of care is an SpO_2 of 96% or above. Small pilot studies have demonstrated that permissive hypoxaemia (aiming for a lower SpO_2 than normal by using a lower fractional inspired oxygen concentration (F_IO_2)) can be achieved in the critically ill and appears to be safe. This approach has not been evaluated in a National Health Service setting. It is possible that permissive hypoxaemia may be beneficial to critically ill patients thus it requires robust evaluation.

Methods and analysis: Targeted OXygen therapY in Critical illness (TOXYC) is a feasibility randomised controlled trial (RCT) to evaluate whether recruiting patients to a study of permissive hypoxaemia is possible in the UK. It will also investigate biological mechanisms that may underlie the links between oxygenation and patient outcomes. Mechanically ventilated patients with respiratory failure will be recruited from critical care units at two sites and randomised (1:1 ratio) to an SpO_2 target of either 88-92% or ≥ 96% whilst intubated with an endotracheal tube. Clinical teams can adjust FIO_2 and ventilator settings as they wish to achieve these targets. Clinical information will be collected before, during and after the intervention and blood samples taken to measure markers of systemic oxidative stress. The primary outcome of this study is feasibility, which will be assessed by recruitment rate, protocol adherence, and withdrawal rates. Secondary outcomes will include a comparison of standard critical care outcome measures between the two intervention groups, and the measurement of biomarkers of systemic oxidative stress. The results will be used to calculate a sample size, likely number of sites, and overall length of time required for a subsequent large multicentre RCT.

For peer review only - http://bmjopen.bmj.com/site/about/guidelines.xhtml

Ethics and dissemination: This study was approved by the London - Harrow Research Ethics Committee on 2nd November 2017 (REC Reference 17/LO/1334) and received HRA approval on 13th November 2017. Results from this study will be disseminated in peer reviewed journals, at medical and scientific meetings, in the NIHR Journals Library and patient information websites.

The study has been funded by the National Institute of Health Research and Royal Free Charity. It is registered on www.clinical trial.gov (NCT03287466). The sponsor is University College London.

STRENGTHS AND LIMITATIONS OF THIS STUDY

- This is the first study of permissive hypoxaemia in critically ill patients in a National Health Service setting
- It is a small randomised controlled trial to assess feasibility, not the efficacy of the intervention
- The study will compare levels of biomarkers of systemic oxidative stress between the two intervention groups
- It will provide valuable information to enable the design of future large-scale randomised controlled trials

For peer review only - http://bmjopen.bmj.com/site/about/guidelines.xhtml

INTRODUCTION

In the UK there are over 190,000 admissions to adult critical care units each year (www.icnarc.org). Approximately 40% of these patients will require mechanical ventilation and the mortality rate in this group is approximately 30%.[1, 2] Hypoxaemia is common amongst this cohort of patients and we lack evidence-based guidelines for their management, particularly regarding what levels of arterial oxygenation are acceptable or optimal. It has been proposed that attempting to fully reverse hypoxaemia in critically ill patients may pose a greater risk of harm than allowing moderate hypoxaemia to persist, a concept called permissive hypoxaemia.[3] The premise behind permissive hypoxaemia is that the interventions used to correct hypoxaemia may themselves cause harm, in particular high concentrations of inspired oxygen, therefore safely minimising their use could be beneficial.[4–7]

Oxygen has the potential to cause harm when used in high concentration, primarily via its toxic effect on the lungs.[8–10] Reactive oxygen species (ROS) released mainly from the inner mitochondrial membrane during oxidative phosphorylation serve an essential role in cellular signalling but in excess these highly reactive molecules are able to destroy lipids, proteins and DNA. Their rate of release is determined by cellular oxygen tension[11] and the extent of damage caused by them can be measured by evaluating biomarkers of tissue degradation[12]. The lung parenchyma is particularly susceptible to oxygen toxicity in critically ill patients as a result of being exposed to high concentration oxygen during mechanical ventilation. The threshold above which harm may be caused (in terms of concentration and duration of exposure) in critically ill patients is unclear, but since lung injury is common in this patient cohort the threshold may well be lower than in other patients or healthy volunteers. During critical illness the propagation of pro-inflammatory pathways, with the activation of leukocyte and vascular endothelial responses further increase the ROS burden.[13] This depletes endogenous antioxidants, which normally

For peer review only

For peer review only - http://bmjopen.bmj.com/site/about/guidelines.xhtml

regulate ROS homeostasis.[14] As a consequence, oxidative stress is a key mechanism of injury in systemic multi-organ failure, and has been linked to increase in morbidity and mortality in critical illness. [15, 16]

Oxygen is a drug a relatively narrow therapeutic index. It should therefore be prescribed, administered and monitored in a manner comparable to other drugs that have toxic side effects. There appears, however, to be wide variation in practice regarding its use and opinions about oxygenation in the critically ill. [17, 18] This is perhaps the result of a paucity of evidence from robust clinical trials; a somewhat surprising situation given that almost every patient admitted to a critical care unit will receive supplementary oxygen. The traditional teaching that hypoxaemia must be avoided at all costs, may have led to a disregard to the potential harm caused by excessive oxygen, and this requires evaluation.

A small number of studies have begun to explore permissive hypoxaemia as a viable treatment strategy in the critically ill, primarily assessing feasibility and safety. In the first study of its kind, 105 mechanically ventilated patients were assessed in a before (n=51) and after (n=54) design. [19] Following a period of standard oxygenation practice in a single centre (aiming for normal or high blood oxygen levels), a practice change was initiated in which oxygen saturation (SpO_2) was maintained at 90–92%. The authors of this study concluded that the conservative oxygen therapy intervention was feasible and free of adverse biochemical, physiological, or clinical outcomes. A comparable strategy was used on a much larger scale in a two-stage model, moving from normal oxygenation to an SpO_2 of 92–95%.[20] These authors reported that mechanical ventilation time was significantly lower during both study (lower SpO_2) phases compared to baseline. The adjusted ICU mortality and ICU-free days did not significantly differ between study phases but mortality decreased in reference to baseline for both of the low SpO_2 phases. In the first multicentre randomised controlled trial (RCT) of permissive hypoxaemia a

For peer review only - http://bmjopen.bmj.com/site/about/guidelines.xhtml

total of 103 mechanically ventilated patients were allocated to either a conservative oxygenation group (SpO_2 of 88–92%) or a liberal oxygenation group (SpO_2 of greater than or equal to 96%.[21] The purpose of the study was to confirm feasibility and this was demonstrated, along with no excess of adverse events in the low SpO_2 group. The most recently published trial of permissive hypoxaemia was a single centre RCT that compared SpO_2 targets of 94%-98% versus 97-100%.[22] The primary outcome of this study was ICU mortality, and the values reported were: 11.6% in the conservative group and 20.2% in the conventional group, giving an absolute risk reduction of 8.6% (1.7-15.0%). This study had a number of limitations[23] but still adds weight to the argument that permissive hypoxaemia appears to not be harmful and may be of benefit.

A factor of great importance to the design of future studies is selecting the correct 'standard' treatment group, in order that the comparison to an intervention of lower oxygenation is valid and meaningful. Different studies have approached this in different ways (either by aiming for an oxygenation target or by determining the administered concentration of oxygen). We hope this feasibility will evaluate our selected methodology and allow us to compare it to other approaches that have been used. We also hope the results of this study will allow us to understand more about other issues specific to critically ill patients such as the concomitant presence of anaemia, low cardiac output and acute respiratory distress syndrome and chronic obstructive pulmonary disease.

METHODS

The trial was designed according to the SPIRIT (Standard Protocol Items: Recommendations for Interventional Trials) statements.[24]

For peer review only - http://bmjopen.bmj.com/site/about/guidelines.xhtml

Trial aim and objectives

The Targeted OXYgen therapy in Critical illness (TOXYC) study aims to determine whether reducing the SpO_2 target in patients requiring mechanical ventilation is feasible (in terms of participant recruitment and delivery of the intervention) in a National Health Service (NHS) setting. In doing this we hope to inform future investigators who wish to construct larger trials in this field. The objectives are to construct a randomised controlled trial of conventional oxygenation versus permissive hypoxaemia, identify any potential barriers to research in this field and explore biological mechanisms that may explain the proposed benefits from the intervention. The project was favourably supported at the UK Critical Care Research Forum in 2016.

Primary outcome measure

The primary outcome of this study is feasibility, which will be assessed in the following ways: i) the ability to recruit patients (recruitment rate), ii) protocol deviations, iii) rate of withdrawal from the study (in both the intervention and control groups), and iv) the reasons for any withdrawal from the study.

Feasibility of recruitment will be evaluated by monitoring patient screening and subsequent agreement to participate, along with any withdrawal of consent during or after the study. Implementation of the study protocol will be evaluated by analysing adherence to oxygenation targets and completion of the treatment without protocol deviations. Reasons for withdrawal will be assessed by the trial management group at the end of the study to assess whether there are common themes that can be addressed in the future.

Secondary outcome measures

Measurements of oxidative stress (including 4- hydroxynonenal, protein carbonyls, total antioxidant capacity and glutathione reductase) will be made in blood samples taken from

For peer review only

participants to understand the potential biological mechanisms that link blood oxygen levels to clinical outcomes. In addition, routine clinical data and outcome measures will be collected from the participants to assess any adverse effects caused by the intervention. Finally, length of critical care stay, length of hospital stay, and survival at critical care unit discharge, 30 and 90 days will be collected. This information will be essential for the design of future larger trials.

Trial design

TOXYC, a multi-centre RCT, which will be conducted at two sites, is a trial of targeted oxygen therapy in adult critically ill patients receiving mechanical ventilation via an endotracheal tube. Sixty patients will be allocated on a 1:1 basis to either a normal SpO_2 target group or a lower than normal SpO_2 target group. A flow diagram of the study is shown in Figure 1. Recruitment began in February 2018 and is planned to continue for 15 months.

Selection of participants

Screening

Screening will occur as part of routine research activity on the two critical care units involved in the study. Research nurses will use medical notes to determine initial suitability for the study, according to the inclusion and exclusion criteria. No additional tests or examinations will be required to ascertain whether patients are eligible for the study. Screening will occur as patients are admitted to the critical care units to minimise the time from admission to enrolment.

Inclusion criteria

- Unplanned admission to a critical care unit
- 18 years of age and above (no upper age limit)
- Respiratory failure forms part of the admission diagnosis
- Enrolled within 24 hours of admission (if already intubated) or within 24 hours of intubation (if intubated on ICU)

For peer review only - http://bmjopen.bmj.com/site/about/guidelines.xhtml

- The patient is expected to receive mechanical ventilation for > 72 hours

Exclusion criteria

- Admission following surgery (elective or unplanned)
- Those patients expected to die within 24 hours of admission to ICU
- Pregnant females
- Admission post-cardiac arrest
- Patients with chronic lung disease known (or highly suspected) to have baseline oxygen saturations in the range of the intervention arm (i.e. 88-92%)
- Admission post trauma (including traumatic brain injury)
- Known sickle cell trait or disease
- Ongoing significant haemorrhage or profound anaemia
- Severe peripheral vascular disease
- Severe pulmonary hypertension
- Other medical conditions where mild hypoxaemia would be contra-indicated
- Patients participating in other interventional clinical trials

Enrolment

Consent

Due to the severity of illness of the patients being recruited to this study, and the use of sedative drugs that are required for mechanical ventilation, it is unlikely that potential participants will have capacity. Should a potential participant be deemed to have capacity they will be approached by the research team, given a patient information sheet (PIS) and then provided with an opportunity to ask questions. After an appropriate length of time, the research team will seek informed consent from the patient if they wish to participate.

For peer review only - http://bmjopen.bmj.com/site/about/guidelines.xhtml

For peer review only

If the patient lacks capacity to provide informed consent, a Personal Consultee (PeC) will be appointed to represent them. This could be the patient's next of kin, a relative or close friend with whom to discuss the patient's participation in the trial. The research team will seek the PeC's opinion as to whether patient would wish to take part in the trial, providing for them an appropriate version of the ethically approved PIS. If the PeC believes the patient would have wanted to participate in the study (or would not have objected to it), they will be asked to sign a PeC agreement form. If there is no PeC present or immediately available in person, opinion may be sought from a suitable person via the telephone and then a telephone agreement form completed by a member of the research team.

If there is no identifiable PeC for a potential participant then they will be provided with a Professional Consultee (PrC), who is completely independent of the study. Their opinion will be sought as to whether it is appropriate for the patient to be enrolled into the study. Opinion will be sought in the same manner as for the PeC, involving the appropriate version of the ethically approved PIS.

Adequate time will be given for consideration by the patient or PeC/PrC to consider the information in the PIS and ask questions. The research team will record when the PIS has been given to the patient or their consultee. Due to the nature of this patient cohort (critically ill patients requiring substantial organ support due to the severity of their illness) the length of time from identifying a potential participant to initiating the intervention is likely to be less than 24 hours. This is to avoid dilution of the intervention or control effect prior to its commencement.

If a participant who lacked capacity at the point of recruitment subsequently becomes able to provide informed consent (because they gain capacity on recovery from their illness), they will be informed about their participation in the study, provided with a PIS and asked whether they would be willing to provide retrospective consent. At this point the participant will be given the

For peer review only - http://bmjopen.bmj.com/site/about/guidelines.xhtml

opportunity to withdraw from the study and to decide if the data (and blood samples) collected from them can be included in the final analysis.

All patient consent and consultee agreement procedures will adhere to the Mental Capacity Act (2005).

Randomisation

Randomisation will be carried out online after a patient has been recruited to the study and an agreement form or consent form has been signed. It will be conducted in a 1:1 manner for the intervention and control groups, stratified by study site, using random permuted blocks of different size. The process of randomisation will be conducted online (www.sealedenvelope.com).

Withdrawal

Participants will be withdrawn from the study if:

- The responsible clinician deems it inappropriate for treatment to continue due to a change in the patient’s condition
- The Chief Investigator or delegated member of the research team deems it inappropriate for treatment to continue due to a change in the patient’s condition
- Agreement for the patient to participate in the study is withdrawn by the PeC or PrC
- The patient regains capacity and choses to withdraw from the study

As this is a feasibility study, patients withdrawn from the study will not be replaced, but the reason for withdrawal will be recorded.

For peer review only

Trial treatment

Intervention

The intervention is a more conservative use of oxygen via the ventilator to achieve an SpO_2 of 88-92%, lower than normal practice in most critical care units in the UK. The intervention will be delivered by the participant's clinical team, which will consist of the critical care doctors and nurses at the two study centres. These teams will be provided with guidance to help keep participants within their target SpO_2 (*supplementary material 1*) but this will not be protocolised. Due to the nature of the intervention, neither the research or clinical teams can be blinded to participant group allocation.

Comparator

The control group will receive oxygen to maintain an SpO_2 at 96% or above (standard care). As per the intervention group, guidance will be provided to the clinical team to help maintain participants within their target SpO_2 (*supplementary material 2*).

Duration of treatment

The aim is for the intervention to be commenced as soon as possible after admission to the critical care unit (following enrolment) and end following removal of the participant's endotracheal tube. Specific treatment end-points for both groups would therefore include i) extubation, ii) formation of a tracheostomy, iii) transfer to another critical care unit, and iv) death. The research team will review enrolled participants daily to monitor adherence to SpO_2 targets and provide bedside advice where required. No targets or limitations will be set for arterial partial pressure of oxygen (PaO_2) or carbon dioxide ($PaCO_2$). Should a patient in the study be transferred out of the ICU for a short period of time (e.g. for an investigation or intervention) the protocol will be paused until their return. Whilst out of the ICU the clinical team will be in control of the patient's oxygenation. Should a patient's condition deteriorate to such an extent that

For peer review only

either the clinical or research team feel it not in the patient's best interest to continue in the study, they will be withdrawn from it at that point.

Standard clinical management.

Aside from the designated SpO_2 targets, all other aspects of care will remain the same between the intervention and control groups. Regular arterial blood gases should be taken during the trial period, according to local clinical guidelines; no additional arterial blood gases will be necessary for the purpose of the study.

Data collection

Data will be collected from various sources including the participant's medical records, bedside charts and hospital computer systems. Data will be collected from these primary sources by members of the research team and entered into an electronic clinical record form (eCRF).

Baseline data collection

- Patient demographics: age, gender, height and weight
- Cause of respiratory failure (diagnosis)
- The presence of any chronic diseases
- Acute Physiology and Chronic Health Evaluation (APACHE) II score (and its components)
- Sequential Organ Failure Assessment (SOFA) score (and its components)
- *Respiratory measurements*: PaO_2, $PaCO_2$, pH, SpO_2, FIO_2, ventilator settings and measures
- *Cardiovascular measurements*: blood pressure, heart rate, cardiac rhythm, vasopressor / inotrope dose, fluid balance
- *Renal measurements*: creatinine, urine output in past 24 hours, the need for renal replacement therapy
- *Hepatic measurements*: transaminases, blood clotting values and bilirubin
- Blood lactate concentration

For peer review only - http://bmjopen.bmj.com/site/about/guidelines.xhtml

Subsequent data collection during treatment

- Most measures will be taken daily, except for those specifically related to oxygenation, which will be collected hourly, to permit detailed analysis of compliance to blood oxygenation target.
- Time to extubation or detachment from mechanical ventilation, and mechanical ventilation free days on ICU
- Adverse events occurring during the study period

Follow up

- Length of ICU stay
- Length of hospital stay
- 30 and 90 day survival rates, and days alive out of hospital
- Adverse events

Data Management

This trial will use an eCRF and trial data will be entered into an approved, protected database (https://www.elsevier.com/solutions/macro). Access to the eCRF system will only be provided to staff with relevant authority. Participants will be given a unique subject number and subject identifier. Data will be entered under this identification number onto the central database stored on the servers. The database will be password protected and only accessible to members of the TOXYC study team and external regulators if requested. At site, access will only be granted to staff with permission on the delegation log, and after training. The servers are protected by firewalls and are patched and maintained according to best practice. The physical location of the servers is protected by CCTV and security door access. The database software provides a number of features to help maintain data quality, including; maintaining an audit trail, allowing custom validations on all data, allowing users to raise data query requests, and search facilities

For peer review only

to identify validation failure/ missing data.

The identification, screening and enrolment logs, linking participant identifiable data to the pseudo-anonymised subject numbers will either be held in written form in a locked filing cabinet or electronically in password protected form on hospital computers. After completion of the study the identification, screening and enrolment logs will be stored securely by the sites for 20 years. After completion of the study the identification, screening and enrolment logs will be stored securely by the sites for 20 years.

Sample collection, storage and processing

Blood samples will be taken from participants in order to evaluate oxidative stress. Samples will be taken from an indwelling arterial catheter that is already present in the patient as part of routine critical care. Blood will be processed at each of the two centres according to a defined standard operating procedure, and then stored at -80 degrees centigrade. These blood samples will be taken at baseline (shortly after the patient has been recruited into the study but prior to their treatment being commenced), and on days 2, 3, 5 and 10 after recruitment. A number of biomarkers of oxidative stress will be measured, including: 4- hydroxyl-2-nonenal, protein carbonyls, total antioxidant capacity and glutathione reductase.

Safety monitoring

All adverse events will be recorded in the medical records and reported to the appropriate body; either online to SITU or by email to the sponsor. Table 1 shows a basic list of expected adverse events that will be recorded within the patient's eCRF and medical notes, but the sponsor will not be informed. Adverse events (AEs) and serious adverse events (SAEs) will be summarised descriptively by interventional group at the end of the study.

For peer review only - http://bmjopen.bmj.com/site/about/guidelines.xhtml

RESPIRATORY	CARDIOVASCULAR	HAEMATOLOGICAL
• reintubation<br>• arterial desaturation<br>• pneumothorax<br>• pleural effusion<br>• pneumonia<br>• pulmonary embolism	• arrhythmia<br>• hypotension<br>• requirement for inotropic support	• Anaemia<br>• Low platelet count<br>• High white blood cell count
**RENAL**	**GASTROINTESTINAL**	**NEUROLOGICAL**
• Acute kidney injury<br>• Requirement for renal support<br>• hyperkalaemia	• Diarrhoea<br>• vomiting<br>• Failure to absorb enteral feed	• Delirium / agitation

Table 1. A list of expected adverse events that may occur during the course of the study

For peer review only

Trial monitoring and oversight

The TOXYC trial will report to a data monitoring committee, and a trial steering committee will be appointed to provide study oversight on behalf of the sponsor and funder. Day to day management of the trial will be the responsibility of the trial coordinator with oversight from the trial management group. Permission for protocol amendments will be sought via the sponsor and if deemed necessary the Research Ethics Committee.

Statistical design

No sample size calculation was performed to determine the number of participant required for this trial. The reason for this was that it is a feasibility study in which no formal comparative analyses are planned. The primary and secondary outcome measures will be presented using summary statistics (e.g. means, standard deviations, medians, proportions). Missing data, non-compliers and withdrawals will be looked at in detail to determine if there is any evidence of

For peer review only - http://bmjopen.bmj.com/site/about/guidelines.xhtml

bias.

Those analysing the data will be blinded to specific group allocation. A CONSORT diagram will be completed, summarising the number of patients eligible for the study, the number randomised in each arm, and enumerating in detail those not approached (with reasons), the number of withdrawals (with reasons) overall and per arm. Recruitment rate (overall, per site, and peak recruitment rate per month) will be determined. Monthly and cumulative accrual graphs will be constructed. Baseline characteristics of randomised participants will be summarised, including gender, age, height, weight, details of medical history, pre-intervention APACHE II and SOFA scores. Mean and SD, or median and IQR, will be calculated as appropriate. For the secondary objectives, summary statistics on respiratory, cardiovascular, renal, and hepatic measurements will be calculated at the appropriate time points (hourly or daily). Length of stay in the critical care unit and in hospital will be summarised. 30 and 90 day mortality rates will be calculated, and "days alive and out-of-hospital" determined for each patient and summarised using appropriate measures. Compliance will be assessed. For each patient, the proportion of time spent within the randomisation-determined oxygen saturation limits will be calculated, and summarised by treatment arm. Adverse events will be tabulated and grouped according to seriousness, severity and causality.

All variables will be checked for completeness and checked for the presence of outliers. Graphical depictions of results will be prepared, both on a per-patient basis (especially for the longitudinal data such as hourly oxygen measurement) and grouped by intervention. Frequency distribution curves will be shown where appropriate (especially for the "length of stay" measurements).

No hypothesis testing is envisaged for this feasibility study. The results will be used to calculate a sample size, likely number of sites, and overall length of time required for a subsequent large

For peer review only - http://bmjopen.bmj.com/site/about/guidelines.xhtml

multicentre RCT.

Ethical compliance

This Trial was approved by the London - Harrow Research Ethics Committee on 02NOV17 (REC Reference 17/LO/1334) and received HRA approval on 13NOV17. TOXYC is also registered on www.clinical trial.gov (NCT03287466)

For peer review only

For peer review only - http://bmjopen.bmj.com/site/about/guidelines.xhtml

Patient and public involvement

In 2014, the UK Intensive Care Society published the results of their James Lind Alliance (JLA) Priority Setting Partnership [25]. The aim was to identify and prioritise unanswered questions about adult critical care that are important to people who have been critically ill, their families, and the health professionals who care for them. One of the identified priorities for research was: *"What is the best way of preventing damage to the lungs of patients receiving respiratory support (ventilation)?"* This study addresses this publicly driven need, by assessing a treatment strategy that has the potential to reduce iatrogenic lung injury to patients on a ventilator and therefore improve survival. At the Royal Free Hospital critical care unit we have a growing group of patients and relatives who are willing to assist with the development of research. This group was formed and is managed by our research team at the Royal Free Hospital. A volunteer member of the public from this group agreed to assist us throughout the study, from the application for funding to dissemination of the findings. This person was a co-applicant on the grant application and is an invited member of the Trial Steering Committee. We hope that including a member of the public in the design and delivery of the study will improve the experience of participants in this an future clinical research projects.

CONCLUSION

The results of this feasibility trial will inform researchers about the ability to conduct a study evaluating permissive hypoxaemia in critically ill patients. It will provide information about recruitment rates in UK critical care units and help to identify any barriers to future research. Furthermore, results from oxidative stress marker analysis may highlight biological markers of importance in the pathway between oxygen administration and patient outcomes.

For peer review only - http://bmjopen.bmj.com/site/about/guidelines.xhtml

Funding statement: This work was supported by the National Institute for Health Research (PB-PG-0815-20006) and Royal Free Charity.

Authors' contributions:

DM: Conceived idea, designed study, obtained funding for RCT, obtained funding for mechanistic component, obtained ethics approval, will be CI for study and PI at Royal Free, wrote manuscript, revised manuscript

CB-G: obtained ethics approval, responsible for trial governance, wrote manuscript, revised manuscript

NM: obtained ethics approval, responsible for trial governance, revised manuscript

GJ: obtained funding for mechanistic component, lead for biochemical analysis, revised manuscript

IP: responsible for data collection, revised manuscript

JS: involved in sample processing, revised manuscript

NW: obtained ethics approval, responsible for trial governance, responsible for data analysis and statistics, wrote manuscript, revised manuscript

MMcN: lead research nurse for study, responsible for screening patients and collecting data, revised manuscript

BRO'D: obtained funding for RCT, involved in study design, revised manuscript

MM: Conceived idea, designed study, obtained funding for RCT, revised manuscript

MG: Conceived idea, designed study, obtained funding for RCT, PI at Southampton General Hospital, wrote manuscript, revised manuscript

Competing interests statement:

For peer review only - http://bmjopen.bmj.com/site/about/guidelines.xhtml

DM, MM and MG are directors of a company developing an oxygen delivery device (Oxygen Control Ltd).

DM has received honoraria and consultancy fees from Siemens Healthcare, Masimo, Deltex and Edwards Lifesciences.

MG is the National Specialty Lead for Anaesthesia, Perioperative Medicine and Pain within the UK National Institute of Heath Research Clinical Research Network, an elected council member of the Royal College of Anaesthetists and serves on the board of the Evidence Based Perioperative Medicine (EBPOM) social enterprise and the medical advisory board of Sphere Medical Ltd. MG has received honoraria for speaking and/or travel expenses from Edwards Lifesciences, Fresenius-Kabi, BOC Medical (Linde Group), Ely-Lilly Critical Care, and Cortex GmBH. MG is executive chair of the Xtreme-Everest Oxygen Research Consortium and joint Editor-in-Chief of the journal *Perioperative Medicine.*

MM is a consultant for Baxter, Edwards Lifesciences and Deltex; his University Chair is supported by Smiths Medical; Elected Council Member Royal College of Anaesthetsists; Editorial Board BJA and Critical Care; Founding Editor-in-Chief Perioperative Medicine.

JS, NW, ROD, GJ and MMcN have no competing interests.

This article presents independent research funded by the National Institute for Health Research (NIHR) under its Research for Patient Benefit (RfPB) Programme (Grant Reference Number PB-PG-0815-20006). The views expressed are those of the authors and not necessarily those of the NHS, the NIHR or the Department of Health.

For peer review only

REFERENCES

1. Eastwood G, Bellomo R, Bailey M et al. Arterial oxygen tension and mortality in mechanically ventilated patients. *Intensive Care Med*. 2012;38:91-98.

2. de Jonge E, Peelen L, Keijzers PJ et al. Association between administered oxygen, arterial partial oxygen pressure and mortality in mechanically ventilated intensive care unit patients. *Crit Care*. 2008;12:R156.

3. Martin DS, Grocott MP. Oxygen therapy in critical illness: precise control of arterial oxygenation and permissive hypoxemia. *Crit Care Med*. 2013;41:423-432.

4. Abdelsalam M, Cheifetz IM. Goal-directed therapy for severely hypoxic patients with acute respiratory distress syndrome: permissive hypoxemia. *Respir Care*. 2010;55:1483-1490.

5. Capellier G, Panwar R. Is it time for permissive hypoxaemia in the intensive care unit? *Crit Care Resusc*. 2011;13:139-141.

6. He HW, Liu DW. Permissive hypoxemia/conservative oxygenation strategy: Dr. Jekyll or Mr. Hyde. *J Thorac Dis*. 2016;8:748-750.

7. Oxygen therapy and the Goldilocks principle. [editorial]. J Intensive Care Soc 2017;18(4):279.

8. Jackson RM. Pulmonary oxygen toxicity. *Chest*. 1985;88:900-905.

9. Crapo JD. Morphologic changes in pulmonary oxygen toxicity. *Annu Rev Physiol*. 1986;48:721-731.

10. Davis WB, Rennard SI, Bitterman PB, Crystal RG. Pulmonary oxygen toxicity. Early reversible changes in human alveolar structures induced by hyperoxia. *N Engl J Med*. 1983;309:878-883.

For peer review only

For peer review only - http://bmjopen.bmj.com/site/about/guidelines.xhtml

11. Fandrey J, Frede S, Jelkmann W. Role of hydrogen peroxide in hypoxia-induced erythropoietin production. *Biochem J*. 1994;303:507-510.

12. Crimi E, Sica V, Slutsky AS et al. Role of oxidative stress in experimental sepsis and multisystem organ dysfunction. *Free Radic Res*. 2006;40:665-672.

13. Abilés J, de la Cruz AP, Castaño J et al. Oxidative stress is increased in critically ill patients according to antioxidant vitamins intake, independent of severity: a cohort study. *Crit Care*. 2006;10:R146.

14. Shyur L, Tsung J, Chen J, Chiu C, Lo C. Antioxidant properties of extracts from medicinal plants popularly used in Taiwan. *Int J Appl Sci Eng*. 2005;3:195-202.

15. Mishra V, Baines M, Wenstone R, Shenkin A. Markers of oxidative damage, antioxidant status and clinical outcome in critically ill patients. *Ann Clin Biochem*. 2005;42:269-276.

16. Han JE, Alvarez JA, Staitieh B et al. Oxidative stress in critically ill ventilated adults: effects of vitamin D3 and associations with alveolar macrophage function. *Eur J Clin Nutr*. 2017

17. Eastwood GM, Reade MC, Peck L, Baldwin I, Considine J, Bellomo R. Critical care nurses' opinion and self-reported practice of oxygen therapy: A survey. *Aust Crit Care*. 2011;25:23-30.

18. Eastwood GM, Reade MC, Peck L, Jones D, Bellomo R. Intensivists' opinion and self-reported practice of oxygen therapy. *Anaesth Intensive Care*. 2011;39:122-126.

For peer review only - http://bmjopen.bmj.com/site/about/guidelines.xhtml

19. Suzuki S, Eastwood G, Glassford N et al. Conservative oxygen therapy in mechanically ventilated patients: a pilot before-and- after trial. *Crit Care Med*. 2014;42:1414-1422.

20. Helmerhorst HJ, Schultz MJ, van der Voort PH et al. Effectiveness and Clinical Outcomes of a Two-Step Implementation of Conservative Oxygenation Targets in Critically Ill Patients: A Before and After Trial. *Crit Care Med*. 2016;44:554-563.

21. Panwar R, Hardie M, Bellomo R et al. Conservative versus Liberal Oxygenation Targets for Mechanically Ventilated Patients. A Pilot Multicenter Randomized Controlled Trial. *Am J Respir Crit Care Med*. 2016;193:43-51.

22. Girardis M, Busani S, Damiani E et al. Effect of Conservative vs Conventional Oxygen Therapy on Mortality Among Patients in an Intensive Care Unit: The Oxygen-ICU Randomized Clinical Trial. *JAMA*. 2016;316:1583-1589.

23. Ferguson ND. Oxygen in the ICU: Too Much of a Good Thing. *JAMA*. 2016;316:1553-1554.

24. Chan AW, Tetzlaff JM, Altman DG et al. SPIRIT 2013 statement: defining standard protocol items for clinical trials. *Ann Intern Med*. 2013;158:200-207.

25. Reay H, Arulkumaran N, Brett S. Priorities for future intensive care research in the UK: results of a James Lind alliance priority setting partnership. *Journal of the Intensive Care Society*. 2014;15.4:288-296.

For peer review only

Figure 1. Study flow diagram

For peer review only

For peer review only - http://bmjopen.bmj.com/site/about/guidelines.xhtml

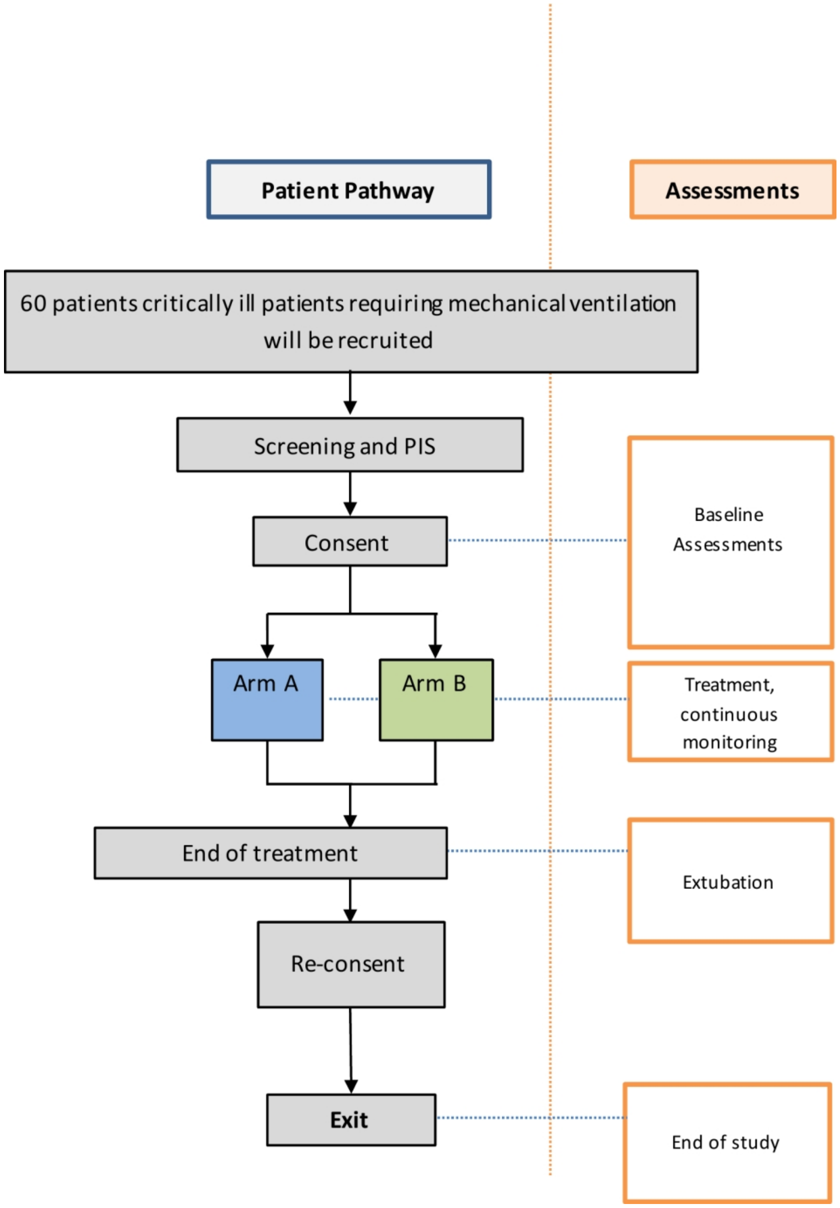

Figure 1. Study flow diagram

127x184mm (300 x 300 DPI)

TOXYC

Guidelines for patients in the **INTERVENTION** group of the **TOXYC** study: **T**argeted **OXY**gen therapy in **C**ritical illness

TARGET SpO_2 RANGE: 88 to 92%

Advice on how to maintain your patient's in the target SpO_2 range for this study:

SpO_2	Advice
> 92%	Reduce FiO_2 in 5-10% intervals every 10 minutes until the SpO_2 is equal to or less than 92%
88-92%	Maintain SpO_2 in target range, reducing or increasing FiO_2 in 5% intervals every 10 minutes if required
< 88%	Increase FiO_2 in 5-10% intervals every 10 minutes until the SpO_2 is equal to or greater than 88%

Other guidance for patients in the **INTERVENTION** group:

- Aim to use the lowest FIO_2 possible to achieve the target SpO_2.
- Try to avoid excessive use of oxygen prior to interventions such as suctioning. Increasing the FIO_2 by approximately 0.25-0.30 (25-30%) briefly should be sufficient in most stable patients.
- Once an FIO_2 of 0.21 (21%) has been reached continue to monitor SpO_2 but no further downwards titration of FIO_2 will be possible.
- Set the SpO_2 alarm limits on the monitor to: LOW = **87%**; HIGH = **93%.**
- Do not adjust the FIO_2 according to the arterial blood gas PaO_2.
- Any mode of ventilation can be used and settings such as the tidal volume, respiratory rate and PEEP can be selected by the patient's clinical team.
- Record all the patient's hourly information in the usual way.
- Please try to minimise unnecessary 100% oxygen boluses and record them all on the ICU chart.

If you have any questions or concerns about the study please contact:
daniel.martin@ucl.ac.uk *or* margaret.mcneil@nhs.net

Thank you for helping us to deliver this study.

Funded by the National Institute for Health Research and Royal Free Charity

NHS
National Institute for Health Research

royal free charity

TOXYC

Guidelines for patients in the **CONTROL** group of the **TOXYC** study: **T**argeted **OXY**gen therapy in **C**ritical illness

TARGET SpO_2 RANGE: ≥ 96%

Advice on how to maintain your patient's in the target SpO_2 range for this study:

SpO_2	
96-100%	Maintain SpO_2 at or above 96% by increasing or reducing F_IO_2 in 5% intervals every 10 minutes if required
< 96%	Increase F_IO_2 in 5-10% intervals every 10 minutes until the SpO_2 is equal to or greater than 96%

Other guidance for patients in the **CONTROL** group:

- Once an F_IO_2 of 0.21 (21%) has been reached continue to monitor SpO_2 but no further downwards titration of F_IO_2 will be possible.
- Set the LOW SpO_2 alarm limit on the monitor to **95%**.
- Do not adjust the F_IO_2 according to the arterial blood gas PaO_2, however, if the SpO_2 is consistently at 100%, care must be taken to avoid unnecessary hyperoxaemia.
- Any mode of ventilation can be used and settings such as the tidal volume, respiratory rate and PEEP can be selected by the patient's clinical team.
- Record all the patient's hourly information in the usual way.
- Please record all 100% oxygen boluses on the ICU chart.

If you have any questions or concerns about the study please contact:
daniel.martin@ucl.ac.uk *or* margaret.mcneil@nhs.net

Thank you for helping us to deliver this study.

Funded by the National Institute for Health Research and Royal Free Charity

NHS National Institute for Health Research

royal free charity

**Administrative information**	
Title	1
	2a
Trial registration	
	2b
Protocol version	3
Funding	4
	5a
Roles and responsibilities	
	5b
	5c
	5d
**Introduction**	
Background and rationale	6a
	6b
Objectives	7

For peer review only

Trial design	8
**Methods: Participants, intervention**	
Study setting	9
Eligibility criteria	10
	11a
	11b
Interventions	
	11c
	11d
Outcomes	12
Participant timeline	13
Sample size	14
Recruitment	15
**Methods: Assignment of interventic**	
Allocation:	

For peer review only

Sequence generation	16a
Allocation concealment mechanism	16b
Implementation	16c
Blinding (masking)	17a
	17b
**Methods: Data collection, managem**	
Data collection methods	18a
	18b
Data management	19
Statistical methods	20a
	20b
	20c
**Methods: Monitoring**	
Data monitoring	21a

For peer review only

	21b
Harms	22
Auditing	23
**Ethics and dissemination**	
Research ethics approval	24
Protocol amendments	25
Consent or assent	26a
	26b
Confidentiality	27
Declaration of interests	28
Access to data	29
Ancillary and post-trial care	30
Dissemination policy	31a
	31b

For peer review only

	31c
**Appendices**	
Informed consent materials	32
Biological specimens	33

For peer review only

Descriptive title identifying the study design, population, interventions, and, if applicable, trial acronym

Trial identifier and registry name. If not yet registered, name of intended registry

All items from the World Health Organization Trial Registration Data Set

Date and version identifier

Sources and types of financial, material, and other support

Names, affiliations, and roles of protocol contributors

Name and contact information for the trial sponsor

Role of study sponsor and funders, if any, in study design; collection, management, analysis, and interpretation of data; writing of the report; and the decision to submit the report for publication, including whether they will have ultimate authority over any of these activities

Composition, roles, and responsibilities of the coordinating centre, steering committee, endpoint adjudication committee, data management team, and other individuals or groups overseeing the trial, if applicable (see Item 21a for data monitoring committee)

Description of research question and justification for undertaking the trial, including summary of relevant studies (published and unpublished) examining benefits and harms for each intervention

Explanation for choice of comparators

Specific objectives or hypotheses

For peer review only

Description of trial design including type of trial (eg, parallel group, crossover, factorial, single group), allocation ratio, and framework (eg, superiority, equivalence, noninferiority, exploratory)

s, and outcomes

Description of study settings (eg, community clinic, academic hospital) and list of countries where data will be collected. Reference to where list of study sites can be obtained

Inclusion and exclusion criteria for participants. If applicable, eligibility criteria for study centres and individuals who will perform the interventions (eg, surgeons, psychotherapists)

Interventions for each group with sufficient detail to allow replication, including how and when they will be administered

Criteria for discontinuing or modifying allocated interventions for a given trial participant (eg, drug dose change in response to harms, participant request, or improving/worsening disease)

Strategies to improve adherence to intervention protocols, and any procedures for monitoring adherence (eg, drug tablet return, laboratory tests)

Relevant concomitant care and interventions that are permitted or prohibited during the trial

Primary, secondary, and other outcomes, including the specific measurement variable (eg, systolic blood pressure), analysis metric (eg, change from baseline, final value, time to event), method of aggregation (eg, median, proportion), and time point for each outcome. Explanation of the clinical relevance of chosen efficacy and harm outcomes is strongly recommended

Time schedule of enrolment, interventions (including any run-ins and washouts), assessments, and visits for participants. A schematic diagram is highly recommended (see Figure)

Estimated number of participants needed to achieve study objectives and how it was determined, including clinical and statistical assumptions supporting any sample size calculations

Strategies for achieving adequate participant enrolment to reach target sample size

›ns (for controlled trials)

Method of generating the allocation sequence (eg, computer-generated random numbers), and list of any factors for stratification. To reduce predictability of a random sequence, details of any planned restriction (eg, blocking) should be provided in a separate document that is unavailable to those who enrol participants or assign interventions

Mechanism of implementing the allocation sequence (eg, central telephone; sequentially numbered, opaque, sealed envelopes), describing any steps to conceal the sequence until interventions are assigned

Who will generate the allocation sequence, who will enrol participants, and who will assign participants to interventions

Who will be blinded after assignment to interventions (eg, trial participants, care providers, outcome assessors, data analysts), and how

If blinded, circumstances under which unblinding is permissible, and procedure for revealing a participant's allocated intervention during the trial

nent, and analysis

Plans for assessment and collection of outcome, baseline, and other trial data, including any related processes to promote data quality (eg, duplicate measurements, training of assessors) and a description of study instruments (eg, questionnaires, laboratory tests) along with their reliability and validity, if known. Reference to where data collection forms can be found, if not in the protocol

Plans to promote participant retention and complete follow-up, including list of any outcome data to be collected for participants who discontinue or deviate from intervention protocols

Plans for data entry, coding, security, and storage, including any related processes to promote data quality (eg, double data entry; range checks for data values). Reference to where details of data management procedures can be found, if not in the protocol

Statistical methods for analysing primary and secondary outcomes. Reference to where other details of the statistical analysis plan can be found, if not in the protocol

Methods for any additional analyses (eg, subgroup and adjusted analyses)

Definition of analysis population relating to protocol non-adherence (eg, as randomised analysis), and any statistical methods to handle missing data (eg, multiple imputation)

Composition of data monitoring committee (DMC); summary of its role and reporting structure; statement of whether it is independent from the sponsor and competing interests; and reference to where further details about its charter can be found, if not in the protocol. Alternatively, an explanation of why a DMC is not needed

Description of any interim analyses and stopping guidelines, including who will have access to these interim results and make the final decision to terminate the trial

Plans for collecting, assessing, reporting, and managing solicited and spontaneously reported adverse events and other unintended effects of trial interventions or trial conduct

Frequency and procedures for auditing trial conduct, if any, and whether the process will be independent from investigators and the sponsor

Plans for seeking research ethics committee/institutional review board (REC/IRB) approval

Plans for communicating important protocol modifications (eg, changes to eligibility criteria, outcomes, analyses) to relevant parties (eg, investigators, REC/IRBs, trial participants, trial registries, journals, regulators)

Who will obtain informed consent or assent from potential trial participants or authorised surrogates, and how (see Item 32)

Additional consent provisions for collection and use of participant data and biological specimens in ancillary studies, if applicable

How personal information about potential and enrolled participants will be collected, shared, and maintained in order to protect confidentiality before, during, and after the trial

Financial and other competing interests for principal investigators for the overall trial and each study site

Statement of who will have access to the final trial dataset, and disclosure of contractual agreements that limit such access for investigators

Provisions, if any, for ancillary and post-trial care, and for compensation to those who suffer harm from trial participation

Plans for investigators and sponsor to communicate trial results to participants, healthcare professionals, the public, and other relevant groups (eg, via publication, reporting in results databases, or other data sharing arrangements), including any publication restrictions

Authorship eligibility guidelines and any intended use of professional writers

For peer review only

Plans, if any, for granting public access to the full protocol, participant-level dataset, and statistical code

Model consent form and other related documentation given to participants and authorised surrogates

Plans for collection, laboratory evaluation, and storage of biological specimens for genetic or molecular analysis in the current trial and for future use in ancillary studies, if applicable

For peer review only

Page

1

4

na

na

4

1

4

na

16,17

5 to 7

5 to 7

18

For peer review only

9

8

9 to 10

12 to 13

12

14

13

8 to 9

Figure 1

17

10 to 12

For peer review only

12

12

12 to 13

13 & 17

na

14 to 16

15

15 to 16

17 to 18

na

17

17

na

16

na

4

17

10 to 12

10 to 12

15

na

15

na

4

na

For peer review only

na

na

16

For peer review only
